# Zeolites as Solid Solvents: Explaining the Chloromethane Hydrolysis over Metal-Exchanged Zeolite Y by DFT Calculations

**DOI:** 10.1021/acsomega.4c03593

**Published:** 2024-09-21

**Authors:** Nilton Rosenbach, Daniella R. Fernandes, Claudio J. A. Mota

**Affiliations:** † Faculdade de Ciência Exatas e Engenharias, Universidade do Estado do Rio de Janeiro, Av. Manuel Caldeira de Alvarenga, 1203, Rio de Janeiro 23070-200, Brazil; ‡ Universidade Federal do Rio de Janeiro, Instituto de Química, Av Athos da Silveira Ramos 149, CT Bloco A, Rio de Janeiro 21941-909, Brazil; § Universidade Federal do Rio de Janeiro, Escola de Química, Av Athos da Silveira Ramos 149, CT Bloco E, Rio de Janeiro 21941-909, Brazil; ∥ INCT Energia & Ambiente, UFRJ, Rio de Janeiro 21941-909, Brazil

## Abstract

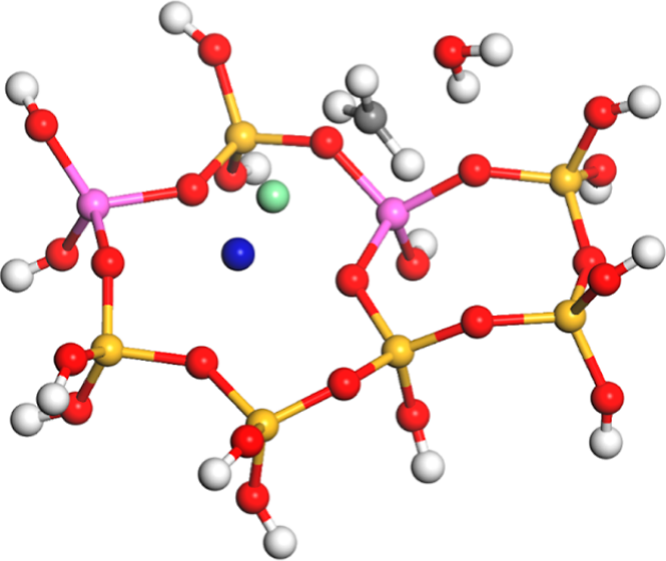

We report a theoretical DFT study of the reaction pathways for chloromethane hydrolysis over metal-exchanged zeolites (Li^+^, Na^+^, K^+^, and Mg^2+^). A cluster of 78 T atoms (T referring to Si or Al atoms), comprising the zeolite Y super cavity coupled with the sodalite cage and three hexagonal prism units (Si_(78–*x*)_Al_*x*_O_129_H_54_; *x* = 1 or 2), was used in the calculations. The study was carried out using the ONIOM method. The high layer was computed at M062X/6-31++G(d,p), whereas the low layer was computed at the PM6 level of theory. The energy profile was obtained by single-point calculations at the M062X/6-31++G(d,p) for the entire structure. The first step studied was the adsorption of chloromethane on the zeolite, which involves an ion–dipole interaction between the metal cation and the chlorine atom. Then, two mechanistic pathways were investigated: one via formation of an adsorbed methoxide and the other involving a direct, one-step, nucleophilic attack of the water molecule to the adsorbed chloromethane. In all systems studied, the direct mechanism showed a lower energy of activation than the route involving the methoxide intermediate. The same behavior was also observed for chloromethane methanolysis to afford dimethyl ether on the metal-exchanged zeolites. The theoretical results are in agreement with the experiments of chloromethane hydrolysis over metal-exchanged zeolite Y, explaining the formation of dimethyl ether upon chloromethane methanolysis on zeolites of low or no acidity. The transition state for the direct route resembles a S_N_2 process assisted by the surface, reinforcing the concept of zeolites as solid solvents, providing a polar nanoenvironment that favors and assists the formation of ionic transition states and intermediates.

## Introduction

The chemistry of C1 mostly encompasses the transformation of methane, the main component of natural and shale gas, and its derivatives.^1^ Methane is also the main component of biogas, which is formed upon the anaerobic fermentation of the organic matter.^2^ The natural gas reforming is the main pathway of methane conversion, yielding syngas, a mixture of carbon monoxide and hydrogen.^3^ Syngas can be used in the Fischer–Tropsch process^4^ to produce gasoline, diesel, and a variety of hydrocarbons. Another route of syngas conversion involves the production of methanol,^5^ which may be further converted to dimethyl ether (DME), a substitute for diesel fuel in compression ignition engines,^6^ as well as olefins (MTO) and hydrocarbons.^7^ Nevertheless, the main issue associated with natural gas reforming is the elevated operational costs, driven by high energy consumption. Roughly, half of the expenses incurred in the so-called gas-to-liquids (GTL) processes stem from the syngas production, impacting the economic feasibility of converting natural and shale gas as well as biogas.

An alternative approach is methane halogenation. For instance, methane chlorination is exothermic by 101 kJ mol^–1^, whereas bromination releases 25 kJ mol^–1^. These reactions can be scaled up for industrial purposes to produce halomethanes, either by direct reaction with molecular halogen (chlorine or bromine) or through oxyhalogenation using HCl or HBr and air. The oxychlorination process typically operates within a temperature range of 350–450 °C. The main inconvenience of both processes is the lack of selectivity due to the free radical mechanism. Thus, to obtain high selectivity in the monohalomethane derivative, an excess of methane must be used. The electrophilic halogenation of methane catalyzed by superacid systems yields monohalomethane with over 99% selectivity.^8,9^ The main drawback is the harsh reaction conditions, also involving highly corrosive media.

**Scheme 1:**
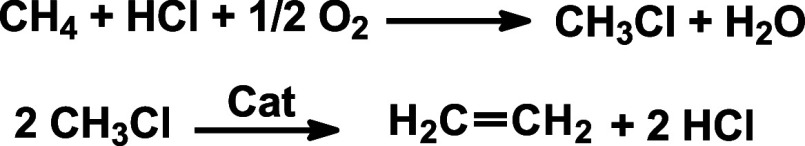
Chlorine-Assisted Conversion of Methane to Ethene

Chloromethane (CH_3_Cl) is a versatile chemical, mostly used in the production of quaternary ammonium salts that may be used as surfactants. Nevertheless, chloromethane can also be converted into hydrocarbons, especially light olefins, such as ethene and propene.^10−12^ This route may compete with the most traditional MTO route, and the hydrogen chloride formed can be recovered for further reuse in methane oxychlorination, turning it into a chlorine-free process in terms of consumption of this feedstock (Scheme 1).

**Scheme 2:**
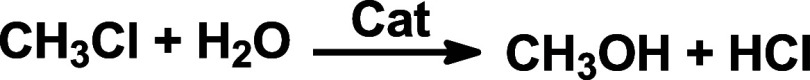
Catalyzed Hydrolysis of Chloromethane to Methanol

Another approach that can be used in the halogen-assisted conversion of methane is the hydrolysis of the monohalomethane to yield methanol (Scheme 2).^13^ A process to convert methane to methanol and DME, involving bromine and metal oxides, has been developed.^14^ It includes the formation of bromomethane as the intermediate, which is subsequently oxidized by O_2_ over the metal oxide catalyst yielding methanol and DME. The oxybromination of methane was also carried out over RuCl_3_ supported on silica.^15^ The subsequent hydrolysis of the bromomethane formed, using a ruthenium complex catalyst, resulted in 98% conversion, yielding 70% of methanol and 30% of DME, after 10 h of reaction.^16^ The catalytic activity of mesoporous γ-alumina in the hydrolysis of chloromethane to methanol and DME has been studied in the temperature range of 170–450 °C.^17^

We have studied the catalytic hydrolysis of chloromethane to methanol and DME over different metal-exchanged zeolite Y.^18,19^ All catalysts showed higher conversions than the blank reaction in the temperature range of 180–400 °C. Methanol is the predominant product, with selectivity ranging from 76 to 95%. DME is the other product formed, and its selectivity depends on the type of cation and acidity of the zeolite catalyst. Hydrocarbons were observed only above 300 °C.

**Scheme 3:**
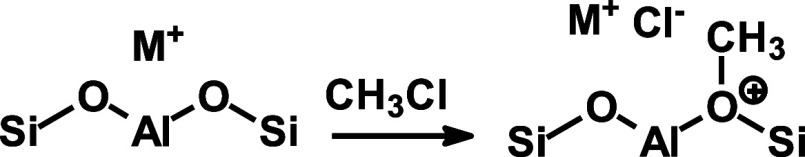
Pictorial Representation of Chloromethane Adsorption over Metal-Exchanged Zeolites to Afford a Methoxy Group and a Metal Chloride Ion Pair

Although the aim of these previous studies was not to investigate the reaction mechanism, it is generally accepted that the initial step of chloromethane conversion over metal-exchanged zeolites is the formation of an adsorbed methoxy species (Scheme 3).^18^ The methoxide intermediate has been spectroscopically detected^20−23^ on the zeolite surface and has been suggested to play a pivotal role in numerous zeolite-catalyzed processes. Therefore, a conceived mechanistic pathway of chloromethane hydrolysis over metal-exchanged zeolites would involve the initial formation of adsorbed methoxy groups, which upon reaction with water affords methanol. In fact, this is the reverse reaction of methanol adsorption over acid zeolites, which has been extensively characterized in the literature.^24−26^ Moreover, it is conceivable that the formed methanol could, potentially, interact with another adsorbed methoxy group to afford DME as a product.

There are few first-principles studies that investigate the interaction between chloromethane and metal-exchanged zeolites. Noronha and collaborators have reported theoretical studies on the interaction of chloromethane with alkaline metal-exchanged zeolites.^12^ According to their findings, the initial step involves the interaction of the chlorine atom with the alkaline metal ion to form an adsorbed methoxy group. In contrast, extensive theoretical studies have focused on the interaction between methanol and acidic zeolites. Despite the structural and chemical similarities between methanol and chloromethane, the conversion of CH_3_Cl to light olefins over the H-SAPO-34 catalyst is slower than that of CH_3_OH under similar reaction conditions.^27,28^ This difference may be attributed to the lower proton affinity of CH_3_Cl (647 kJ mol^–1^) compared to that of CH_3_OH (754 kJ mol^–1^).

To gain insights into the mechanism of methanol formation from the hydrolysis of chloromethane over metal-exchanged zeolites, we conducted a comprehensive DFT-ONIOM study aimed at addressing this inquiry. Hybrid theoretical methodologies, such as QM/MM and ONIOM, have been successfully employed to describe large systems at a computationally feasible cost. This approach is particularly advantageous in the study of catalytic processes involving zeolites, whose structures cannot be adequately treated by using periodic density functional calculations. In the ONIOM scheme, the molecular system can be divided into two or three layers treated at different levels of accuracy.^29^ For example, in the two-layer ONIOM calculations, the atoms of the active site (high layer) can be described with a more sophisticated theoretical level, while the rest of the zeolite structure (low layer) can be treated with less computationally demanding methods. This approach can accurately reproduce the properties of the Bronsted acid sites in ZSM-5 zeolite.^30^ Other studies demonstrate that the ONIOM method can also differentiate crystallographic acid sites, indicating that the lattice plays a significant role on catalytic processes over zeolites.^31^

### Computational Details

**Figure 1:**
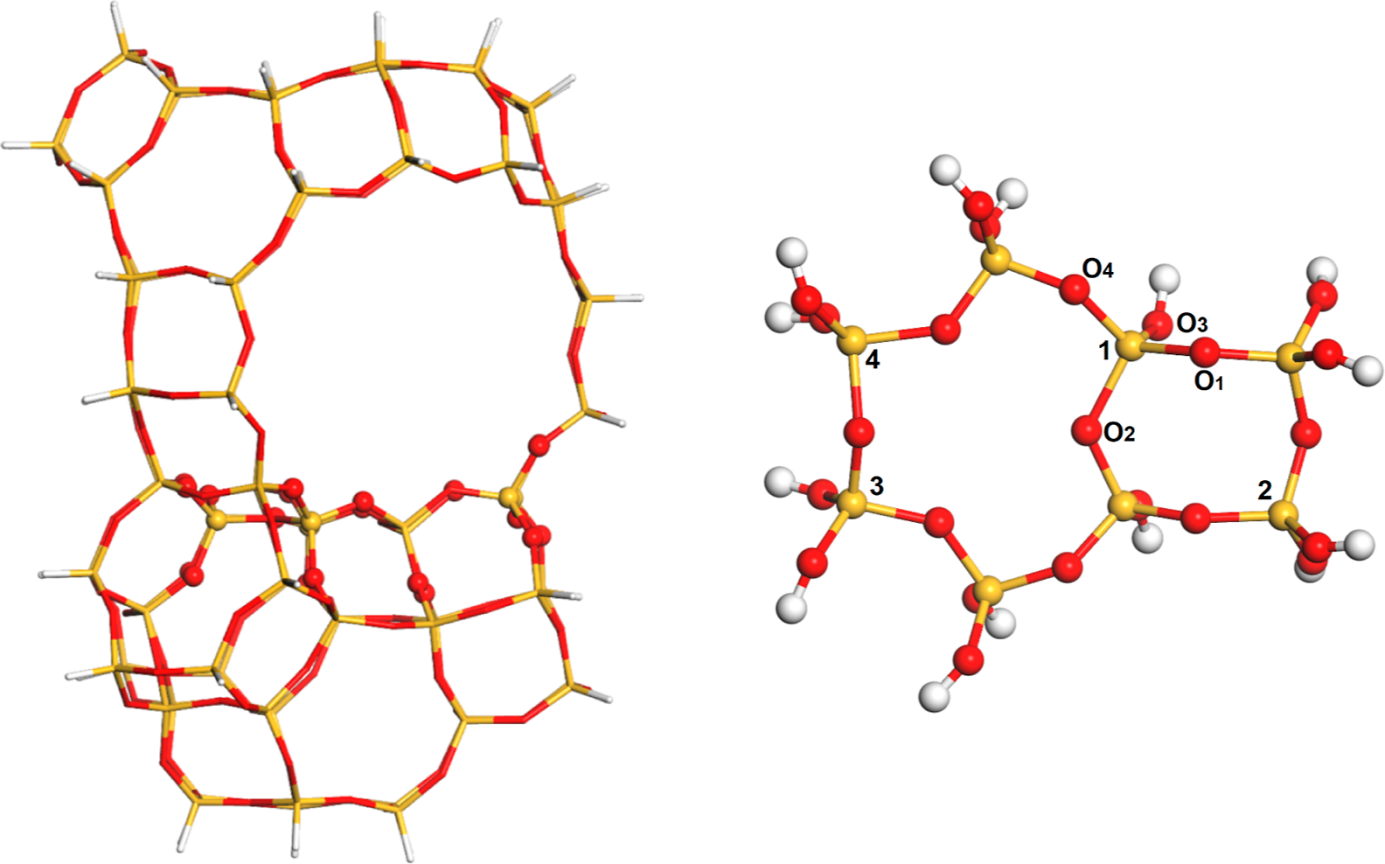
Zeolite Y cluster model used in the ONIOM calculations, highlighting the high-layer (silicon and oxygen) and link (hydrogen) atoms on the right: silicon (yellow), oxygen (red), and hydrogen (white). The numbers denote the positions of the Al atoms in the calculations with two sites. The Al was positioned in position 1 in the calculation with one site.

A cluster model (*T*_78_) of zeolite Y is depicted in Figure 1. It was built up with 288 atoms (Si_(78–*x*)_Al_*x*_O_129_H_54_). This model represents an entire super cavity of the zeolite Y coupled with the sodalite cage and three hexagonal prism units. It was derived from the crystallographic coordinates available in the literature.^32^ To avoid dangling bonds, the free valences of the border silicon atoms were saturated with hydrogen atoms, located at a 1.09 Å distance and in the same plane of the Si–O bond. The position of the hydrogen atoms was kept fixed throughout the optimization steps and vibrational analysis to prevent topological distortion compared with the original zeolite Y structure. Subsequently, aluminum atoms and the organic moiety were introduced into the model. We selected the O1 position to link the organic moiety, because this position is one of the most preferred positions for the proton, according to neutron scattering studies^33^ and theoretical calculations.^34^ Therefore, it is reasonable to assume that other covalent groups would also preferentially occupy this position. Calculations were carried out with Li^+^, Na^+^, K^+^, and Mg^2+^ as counterions. For the divalent Mg^2+^ cation, two framework Al atoms were considered at different relative positions, whereas for the alkaline cations, one framework Al atom was considered in the calculations, except for Li^+^ that was also calculated considering two framework Al atoms. The neutrality of the system was maintained by including an additional Li^+^ ion to neutralize the second framework Al atom.

All calculations were carried out using the ONIOM method implemented in the GAUSSIAN 09 package.^35^ During the optimization steps, the system was divided into two layers (high and low) that were treated at different theoretical levels. The M062X/6-31++G(d,p) method was used to describe the atoms within the high layer, whereas the remaining atoms of the zeolite cavity within the low layer were treated by the semiempirical PM6 method. This method was used with success in similar studies,^36,37^ especially when comparative energy results are the focus of the theoretical work. Single-point calculations at the M062X/6-31++G(d,p) level for the entire structure provided the final electronic energy, incorporating the polarization effect due to the zeolite cavity. Single-point energy calculations were also obtained at the ONIOM[MP2: M062X/6-31++G(d,p)] level. Additionally, Grimme’s empirical dispersion D3 corrections^38^ were also considered in the final electronic energy. Vibrational analysis was carried out for all calculated structures at the ONIOM(M062X/6-31++G(d,p)/PM6) level to take into account zero-point energy (ZPE) corrections and thermal effects (298.15 K). Based on the vibrational analysis, the structures were characterized as either transition states (exhibiting one imaginary frequency mode corresponding to the reaction coordinate in which the C–O bond is formed) or minima on the potential energy surface (absence of imaginary frequencies). Additionally, intrinsic reaction coordinate (IRC) calculations were performed at the same level of theory to ensure that the transition states were accurately connected to the respective minima.

## Results and Discussion

**Figure 2:**
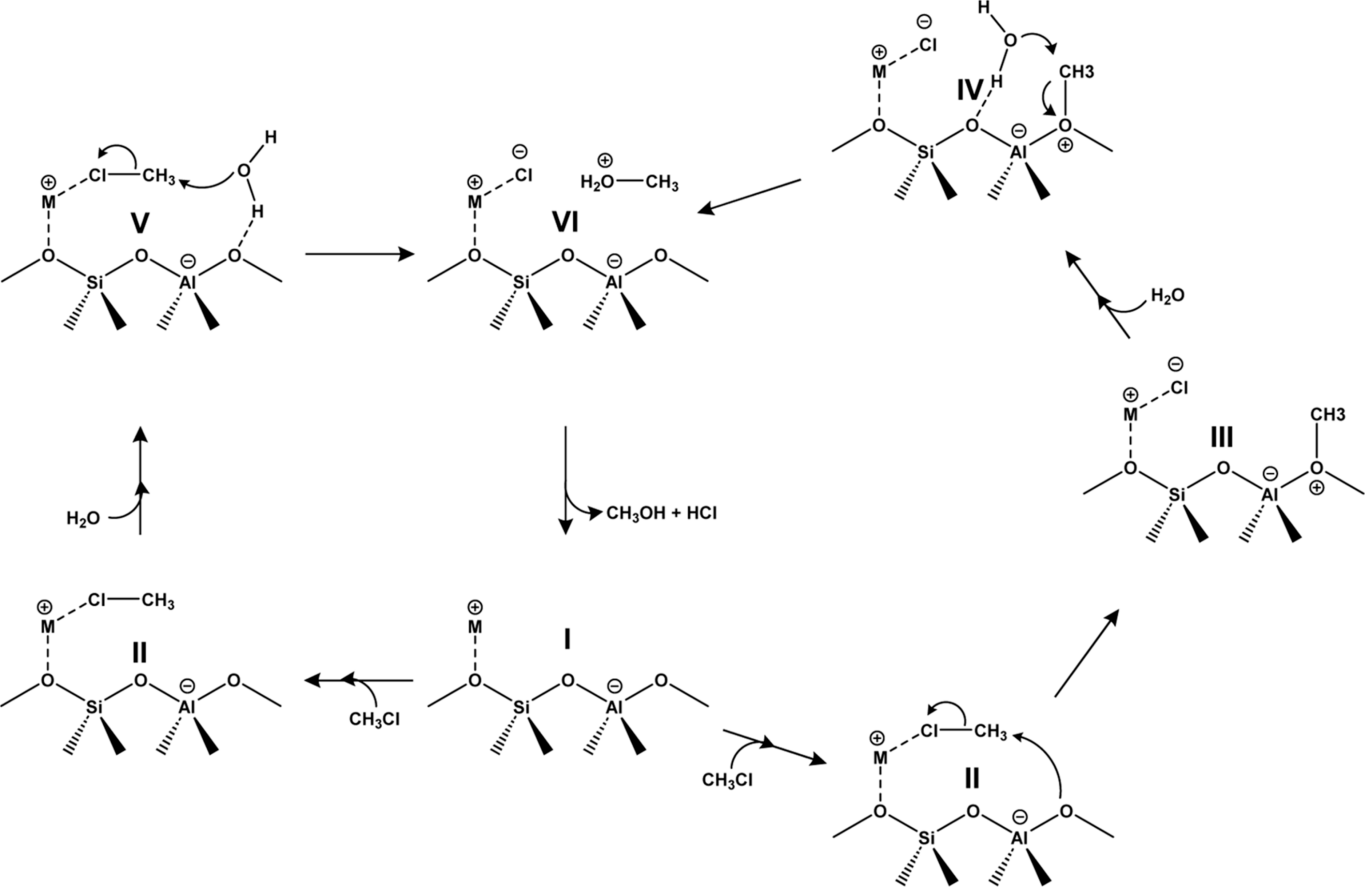
Pathways for chloromethane hydrolysis catalyzed by metal-exchanged zeolite Y: two-step mechanism on the right (I → II → III → IV → VI) and one-step mechanism on the left (I → II → V → VI).

Figure 2 illustrates two possible reaction pathways explored for the hydrolysis of chloromethane over metal-exchanged zeolite Y. The first pathway encompasses two sequential steps. The initial step, after the adsorption of chloromethane, involves the formation of a methoxy intermediate adsorbed onto the surface of the zeolite (structure III). This species is formed through a nucleophilic attack of the framework oxygen atom to the chloromethane molecule, with the simultaneous departure of chloride, assisted by the metal counterion of the zeolite. The subsequent step involves the nucleophilic attack of the formed methoxy intermediate by a coadsorbed water molecule to form protonated methanol (structure VI), which can be desorbed as methanol and HCl, restoring the active site.

The second calculated pathway involves a single step alongside the adsorption of chloromethane and H_2_O and resembles a zeolite-assisted S_N_2 reaction. The adsorbed chloromethane molecule is nucleophilically attacked by a coadsorbed H_2_O molecule, with the simultaneous departure of chloride and formation of protonated methanol, which also desorbs as methanol and HCl. The departure of the chloride ion is also assisted by the metal counterion of the zeolite, as in the two-step mechanism.

We calculated all the species, including the transition states, for both mechanistic pathways on zeolite Y exchanged with monovalent cations like Li^+^, Na^+^, and K^+^, as well as Mg^2+^ representing a divalent cation. The optimized structures of all species within both mechanisms on zeolite Y exchanged with Na^+^ (as illustrated in Scheme 1) at the ONIOM(M062X/6-31++G(d,p)/PM6) level are presented in Figures S1 and S2 of the Supporting Information. The structures of all species in both mechanisms of chloromethane hydrolysis over zeolite Y exchanged with the other cations (Li^+^, K^+^, Cu^+^, and Mg^2+^) are similar. The main differences stem from the radius of the counterion, wherein an increase leads to a slight displacement of the adsorbed species toward the center of the super cage.

Both mechanisms commence with the formation of the adsorption complex, as shown in Figure S1a, resulting from the physisorption of chloromethane. This structure is predominantly characterized by weak electrostatic attractions associated with charge–dipole interactions between the zeolite counterion and the chlorine atom. The physisorption does not significantly disturb the metal position, resulting in a minor displacement of the counterion from its initial position toward the chloromethane molecule (Table S1). Consequently, a slight stretching of the Na–O(zeolite) distances, around 0.1 Å in average, was observed. We did not explore all minima for the adsorption complex inside the zeolite Y pore network since our main goal in this work is to investigate the effect of the counterion on the energy profile of both mechanisms of chloromethane hydrolysis.

**Figure 3:**
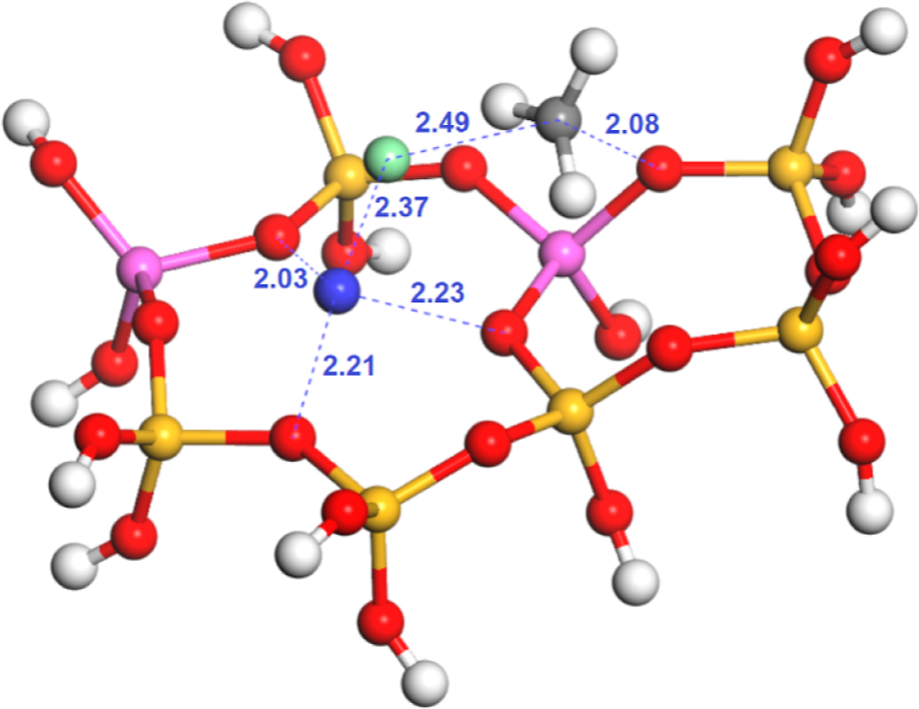
Calculated transition state (TS1) for the methoxide formation from chloromethane over Mg-exchanged zeolite Y (Al atoms in the relative 1–4 position): silicon (yellow), oxygen (red), hydrogen (white), aluminum (pink), carbon (gray), chlorine (green), and magnesium (blue). Distances in Å.

The two-step mechanism proceeds through a surface methoxy group (intermediate III), which is formed through transition state TS1 depicted in Figure 3. In this transition state, the framework oxygen atom attacks the carbon atom from one side, while the chloride anion simultaneously departs from the opposite side, affording the methoxide intermediate and a metal chloride ion pair. The structural characteristics of this type of transition state have been extensively investigated in previous studies, focused on the formation of alkoxide species on the zeolite surface from alcohols and alkyl halides.^12,39^ Based on the O–C distances of the methoxide, summarized in Table S2 of the Supporting Information, our conclusions are similar to the previous findings. The O–C distance in TS1 varies with respect to the metal involved, with Mg^2+^ showing the largest distances, whereas K^+^ presented the shortest. The results suggest that Mg^2+^ shows an earlier transition state, with a less degree of nucleophilic assistance, and K^+^ shows a later transition state, more dependent on the nucleophilic assistance from the framework oxygen atom.

**Figure 4:**
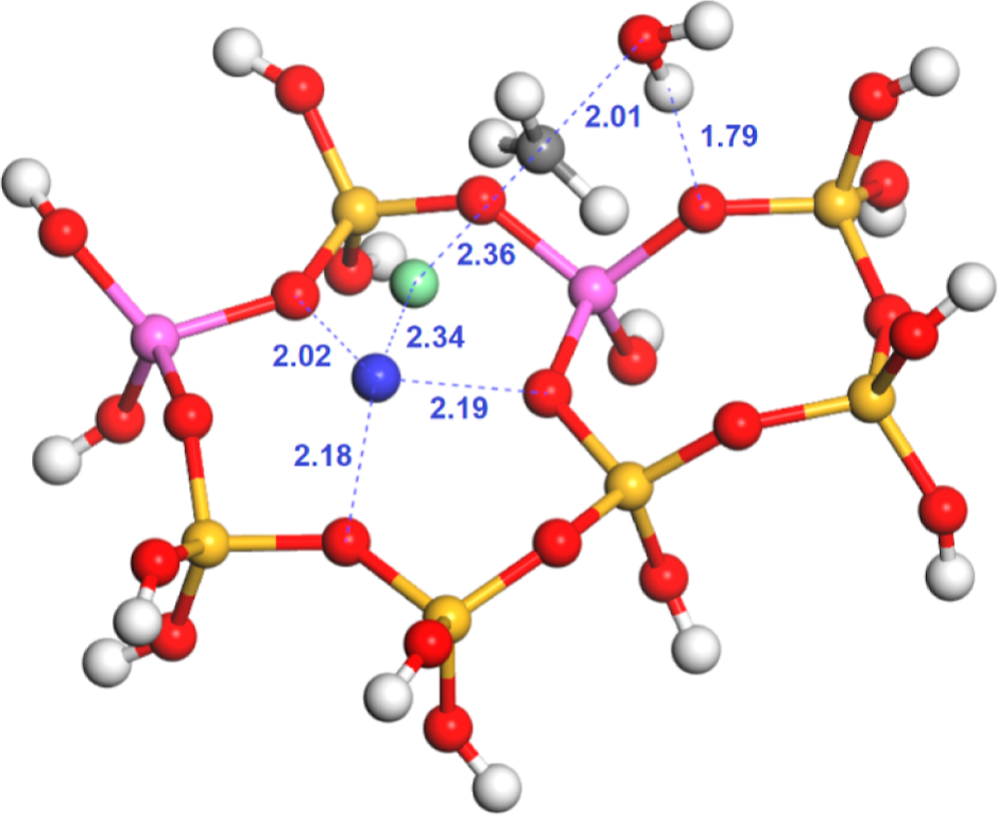
Calculated transition state (TS3) for the direct hydrolysis of chloromethane over Mg-exchanged zeolite Y (Al atoms in the relative 1–4 position): silicon (yellow), oxygen (red), hydrogen (white), aluminum (pink), carbon (gray), chlorine (green), and magnesium (blue). Distances in Å.

The subsequent step involves the adsorption of a water molecule onto the zeolite surface or alkoxide site (intermediate IV). The adsorption of water does not significantly impact the structure of the previously formed methoxide (intermediate III), as evidenced by the geometric data presented in Table S4 of the Supporting Information The most significant change is observed in the position of the chloride ion, which forms a hydrogen bond with the water molecule. Thereafter, the oxygen atom of the water molecule nucleophilically attacks the methoxide to form protonated methanol on the zeolite surface (intermediate V), through transition state TS2 depicted in Figure S1e. In the one-step mechanism, the adsorption of chloromethane (intermediate II) is followed by the coadsorption of a water molecule, forming the adsorption complex illustrated in Figure S2b (intermediate VI). Subsequently, the oxygen atom of the water molecule attacks the chloromethane molecule, similar to a S_N_2 mechanism, leading to the formation of protonated methanol on the zeolite surface (intermediate VI) through transition state TS3 depicted in Figure 4.

**Figure 5:**
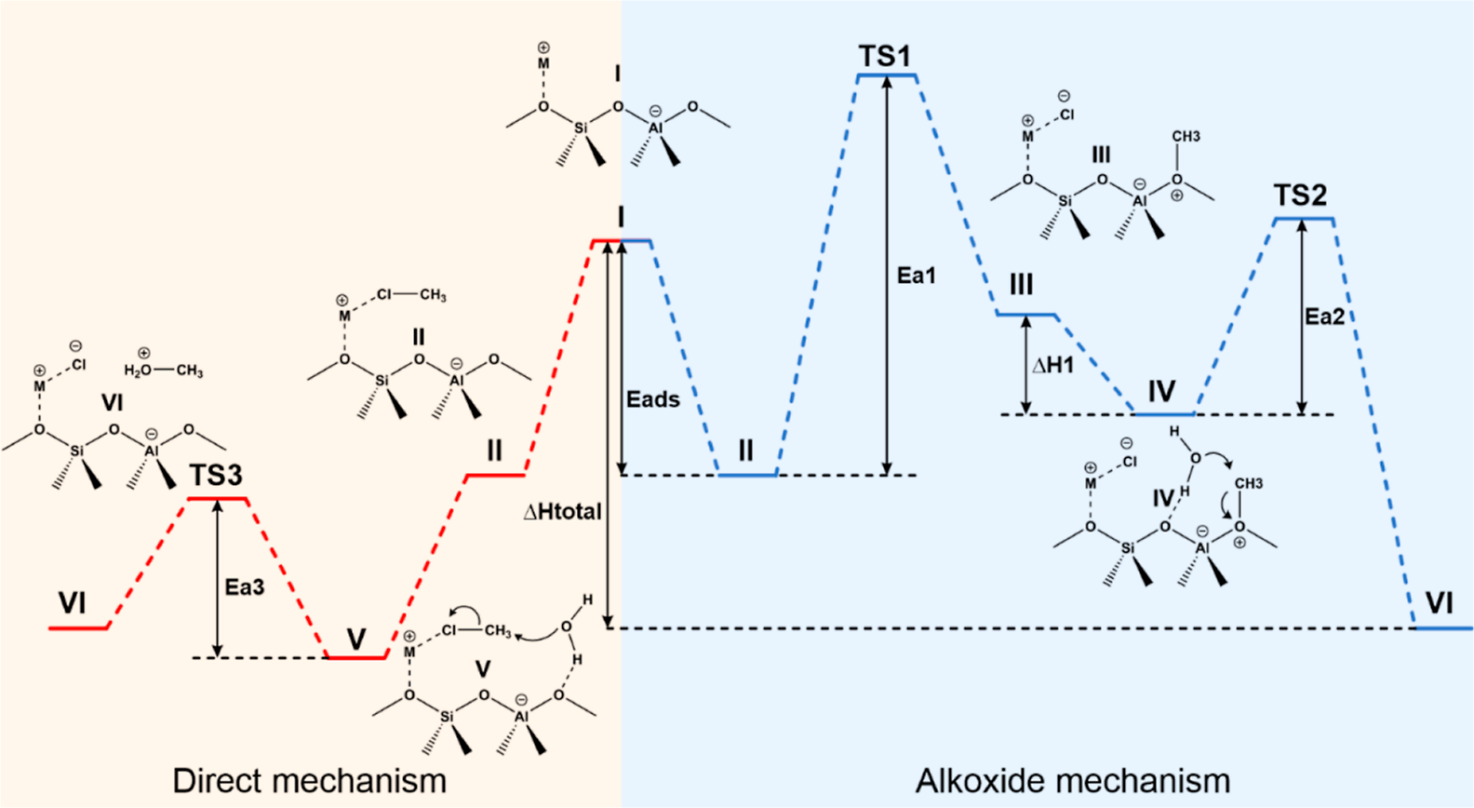
ONIOM-DFT calculations of the energy profiles for the hydrolysis of chloromethane over metal-exchanged zeolite Y, depicting the alkoxide mechanism (blue) and direct mechanism (red).

The energy profile of the hydrolysis of chloromethane over metal-exchanged zeolite Y is depicted in Figure 5 for the two studied mechanisms. The first step in both pathways is the adsorption of chloromethane over the zeolite active site (II), whereas the final calculated state is the protonated methanol and a metal chloride ion pair (V). Table 1 shows the calculated energy profile at M062X/6-31++G(d,p) for the entire structure (high and low layers) for the different zeolite systems considered in this study.

**Table 1:** Calculated Energy Profile, at M062X/6-31++G(d,p) for the Entire Structure High and Low Layers, of the Hydrolysis of Chloromethane over Metal-Exchanged Y Zeolites Considering the Alkoxide and the Direct Route^a^

ion	*E*_ads_	alkoxide route^b^			direct route^b^	Δ*H*_total_
		*E*_a1_	Δ*H*1	*E*_a2_	*E*_a3_	
Hydrolysis						
Li^+^^c^	–69.6	143.9	–55.0	28.4	78.6	–139.5
Li^+^^d^	–73.3 (−75.8)^e^	89.5 (142.8)^e^	–69.5 (−73.6)^e^	115.6 (141.8)^e^	60.1 (85.2)^e^	–166.9 (−154.4)^e^
Na^+^^c^	–65.7	132.3	–38.1	20.3	117.3	–134.0
K^+^^c^	–46.4	126.4	–27.6	23.2	142.7	–118.1
Mg^2+^^d^	–135.4 (−136.9)^e^	98.8 (137.9)^e^	–24.4 (−21.6)^e^	73.6 (92.7)^e^	38.0 (65.4)^e^	–253.8 (−173.8)^e^
Mg^2+^^f^	–105.5	127.7	–41.4	40.4	43.9	–179.7
Mg^2+^^g^	–109.1	120.6	–76.6	76.5	39.4	–217.7
Methanolysis						
Li^+^^c^	–73.3 (−75.8)^e^	89.5 (142.8)^e^	–78.9 (−74.4)^e^	104.5 (119.8)^e^	53.2 (78.7)^e^	–151.7 (−98.6)^e^
Mg^2+^^c^	–135.4 (−136.9)^e^	98.8 (137.9)^e^	–25.6 (−23.8)^e^	71.6 (70.0)^e^	24.1 (57.5)^e^	–234.9 (−218.8)^e^

a The energy values (in kJ mol^–1^) refer to the barriers and energy differences displayed in Figure 5.

b Referred to Figure 2.

c Calculated with 1 Al atom.

d Calculated with 2 Al atoms at 1,2 relative positions (Figure 1).

e Single point energy calculations at the ONIOM(MP2:M062/6-311++G(d,p)) level.

f Calculated with 2 Al atoms at 1,3 relative positions (Figure 1).

g Calculated with 2 Al atoms at 1,4 relative positions (Figure 1).

The results show that adsorption of chloromethane over all metal-exchanged zeolite Y is exothermic compared with the isolated reactants and reflects the strength of the ion–dipole interaction. Throughout the entire simulation series, one can see that the hardest Mg^2+^ cation exhibits higher adsorption enthalpy, with some difference depending on the relative position of the framework Al atoms. It is interesting to note that when two framework Al atoms were considered for the calculation with Li^+^ as the counterion, the adsorption of chloromethane was slightly favored compared with calculations with one site. This finding may be explained by some additional electrostatic stabilization of the adsorbed species by the presence of adjacent ions. We also computed the adsorption of water on LiY and found a value of −84.9 kJ mol^–1^, which is 15.3 kJ mol^–1^ lower in energy than the computed adsorption of chloromethane over the same zeolite system. Considering that the experimental results were obtained at 200 °C and higher temperatures, one must assume that water adsorption is reversible and does not poison the active site of chloromethane hydrolysis, as experimentally observed.

The process is exothermic, yielding protonated methanol on the zeolite surface and a metal halide ion pair. The calculations with Li^+^ as the counterion show that the presence of a second active site stabilizes the final state. The calculations with Mg^2+^ showed a dependency of the Δ*H*_total_ with the relative positions of the Al atoms. It is worth mentioning that we did not explore all minima for the protonated methanol^40^ and the metal chloride ion-pair inside the zeolite Y pore network; thus, the values of Δ*H*_total_ should be interpreted with care. Our main goal in this work was not to investigate the overall thermodynamic profile; the main focus was on the mechanisms of chloromethane hydrolysis and methanolysis on the metal-exchanged zeolites, aiming at identifying the preferred mechanistic pathway. We computed the reaction between the MCl ion pair and protonated methanol to yield HCl and methanol as the final products for the LiY zeolite system. Figure S9 of the Supporting Information shows the structures with the proton on the methanol molecule and on the chloride. The first structure (protonated methanol) is about 19.1 kJ mol^–1^ higher in energy relative to the adsorbed HCl and unprotonated methanol at M062X/6-31++G(d,p). All attempts to find the transition state for this proton interchanged failed, suggesting that the energy barrier is small and both species may coexist in equilibrium. Thus, the entire thermodynamic profile of the process should consider this last reaction, but since this was not the main objective of the study, we did not perform the calculation for all the zeolite systems studied, only for LiY. The same trend should be observed for the other systems, with different energy values.

The formation of methoxide (TS1) involves energy barriers (Ea1) ranging from 99 to 144 kJ mol^–1^, as computed in relation to the respective adsorption complex. Apparently, the interaction between the metal cation and the chlorine atom is not sufficient to overcome the low nucleophilicity of the framework oxygen atom. It is interesting to note that the methoxide is not greatly stabilized relative to the adsorption complex (Δ*H*1) when only one framework Al atom is considered. This is particularly evident for Li^+^ as the counterion. The results reinforce the electrostatic stabilization of the formed LiCl species by the presence of additional ions in the structure. The same pattern may be applied for Mg^2+^, but the relative positions between the framework Al atoms may affect the energy difference between the methoxide and the adsorption complex. Indeed, we have observed in previous studies that electrostatic stabilization plays an important role in the formation and stabilization of alkoxides and carbocations on the zeolite surface.^41^

The next step in the mechanism via alkoxide involves the adsorption of water to form methoxide (IV). Table 1 shows the results, which indicate that intermediate IV is more stable than methoxide on all metal-exchanged zeolites considered in this work. Calculations involving two Al sites stabilize even more the intermediate IV with respect to the alkoxide, favoring the interaction of the water molecule with the surface methoxide. The final step is the nucleophilic attack of the water molecule to the methoxide (TS2) to yield protonated methanol and a metal chloride species (V). One can see in Table 1 that TS2 involves a considerably lower energy of activation (*E*_a2_) than TS1, indicating that formation of the adsorbed methoxide is the rate-determining step. Again, the presence of a second framework Al atom stabilizes the transition state, but to a lesser extent compared with TS1. The value of *E*_a2_ was higher than that of *E*_a1_ when placing the framework Al atoms in the relative 1,2 positions for Li^+^ as the counterion. The reason may be due to the approach of the water molecule to the methoxide, which may be impaired by the zeolite framework in this case. Nevertheless, one can see that the overall barrier for hydrolysis is lower compared to the other calculated cases.

The direct, one-step pathway resembles an S_N_2 mechanism, which is widely accepted to occur in solvents. For all zeolite systems studied, this route presented lower energy of activation (*E*_a3_) compared to the alkoxide pathway, indicating that it is the preferred mechanistic pathway for chloromethane hydrolysis over metal-exchanged zeolites. Again, the presence of two Al sites stabilizes the transition state. Thus, for Li^+^ as the counterion, the difference is 18.6 kJ mol^–1^, indicating the beneficial effect of electrostatic stabilization by a second active site. For Mg^2+^ as a counterion, the energy of activation varies with the relative positions of the framework Al atoms. Nevertheless, in all studied cases, the direct route involved significantly lower barrier than the alkoxide mechanism.

We have also calculated the chloromethane methanolysis for the Li^+^ and Mg^2+^ counterions, considering the system with two framework Al atoms at the 1,2 relative positions. The results are also presented in Table 1 and follow the same general trend observed for the hydrolysis. Thus, the direct, one-step, mechanism is preferred compared with the pathway involving the methoxide intermediate. These results may explain the experimental findings^18^ that showed the formation of DME over NaY zeolite of no or little acidity. Thus, in addition to the acid-catalyzed dehydration of methanol, the methanolysis of chloromethane over metal-exchanged zeolites may be an alternative pathway for the production of DME.

We performed single-point energy calculations [MP2/M062/6-311++G(d,p)] for the two mechanistic pathways. Table 1 shows the results for LiY and MgY with the two framework Al atoms at 1,2 relative positions; the complete energy values are reported in Table S6 of the Supporting Information One can see that the energy of adsorption is not greatly affected by the level of calculation, but the absolute barriers for both pathways are higher when computed at [MP2/M062/6-311++G(d,p)]. The general trend is the same, even with the increasing energy difference between the two mechanisms. For instance, the energy difference between the alkoxide and the single-step mechanism of chloromethane hydrolysis is 55.5 kJ mol^–1^ at M062X and 57.6 kJ.mol^–1^ at ONIOM(MP2:M062X) for the LiY system, stressing the preference for the direct hydrolysis route. The gap is even higher for MgY, being 60.8 kJ mol^–1^ at M062X and 72.5 kJ mol^–1^ at ONIOM(MP2/M062X). For chloromethane methanolysis, the energy difference is 51.3 and 64.1 kJ mol^–1^ for LiY at M062X and ONIOM(MP2:M062X), respectively, whereas for MgY, the gap is 74.7 and 80.4 kJ mol^–1^ at M062X and ONIOM(MP2:M062X), respectively. Thus, improving the level of energy calculation does not change the conclusion that the one-step, direct mechanism, is the preferred pathway of the chloromethane hydrolysis over metal-exchanged zeolites. Indeed, a slight increase in the energy difference between the two mechanistic pathways was observed with the single-point calculations at the MP2 level in the high layer.

The calculations pointed out that the direct pathway, resembling an S_N_2 mechanism occurring at the zeolite surface, presents a lower energy of activation than the alkoxide pathway on all metal-exchanged zeolites studied. The transition state resembles the traditional push–pull mechanism, normally invoked for solvent-assisted nucleophilic substitution reactions.^42^ A similar pathway has been proposed for the formation of DME on the acidic MFI zeolite. Experimental and DFT theoretical results^43^ supported a mechanistic pathway involving a direct route, with two adsorbed methanol molecules that interact through a S_N_2-like transition state. Calculations showed that the route involving an adsorbed methoxide intermediate is significantly higher in energy, in agreement with the calculations presented here for chloromethane.

One may define catalysis as the acceleration of a given reaction due to the presence of a substance (a catalyst) that modifies the general mechanistic pathway upon chemical interaction with the reactants, which leads to a lower energy pathway. Thus, catalysis implies a mechanism for the reaction that is different from the general mechanism in the absence of the catalyst. On the other hand, a solvent may also accelerate a given reaction, but not due to a change in the general mechanistic pathway, but rather because it may increase the polarity of the medium or assisting (interacting or solvating) the formation of the transition state. Thus, all the interactions of the solvent molecules with the reactants and transition states are of physical nature, and there is no chemical bonding with the reactants that would lead to a different and less energetic mechanistic pathway.

The behavior of metal-exchanged zeolites in chloromethane hydrolysis to methanol is similar to the behavior of solvents in nucleophilic substitution reactions. In fact, we have already proposed the solvent-like behavior of zeolites in alkyl halide ionization,^37^ halogen switch,^44^ and formation of cyclic organic carbonate upon the reaction of CO_2_ and epoxides.^45^ In all of these reactions, the zeolite surface favors the reaction between nucleophiles present inside the zeolite cage with incipient electrophilic species formed at the active sites, but without changing the general mechanism. In the present study, the same concept was shown but to a reaction involving two added reactants (an electrophile and a nucleophile). A recent theoretical study,^46^ at a similar level of calculation, pointed out the solvent assistance in the formation of the transition state for nucleophilic substitution reactions. All the interactions are of physical nature, similar to what was computed for the direct, one-step pathway for chloromethane hydrolysis over metal-exchanged zeolites, reinforcing the role of zeolites as solid solvents.

## Conclusions

The hydrolysis of chloromethane over metal-exchanged zeolite Y is an alternative route to methanol and DME, avoiding syngas formation from natural gas or other methane-rich feedstock.

Two potential mechanistic pathways were investigated by DFT calculations: the alkoxide route and the direct, one-step, mechanism. All of them begin with adsorption of choromethane on the zeolite active site, which resembles an ion–dipole interaction between the cation and the chlorine atom.

Calculations indicated that the direct, one-step, mechanismis preferred over the alkoxide route, involving lower energy of activation. The direct route resembles a S_N_2 process occurring on the zeolite surface, with the leaving of the chloride ion assisted by the metal cation. The results support the proposal of zeolites as solid solvents, providing an environment for nucleophilic substitution reactions to take place. In the present case, this concept is extended to adsorbed reactants, being of a broader scope.

## Supporting Information Available

Nine figures with the optimized structures of intermediates and transition states and six tables with geometric data and energy profile PDF)

## References

[ref1] Lunsford J. H. Catalytic Conversion of Methane to More Useful Chemicals and Fuels: A Challenge for the 21st Century. Catal. Today 2000, 63 (2–4), 165–174. 10.1016/S0920-5861(00)00456-9.

[ref2] Khan M. U.; Lee J. T. E.; Bashir M. A.; Dissanayake P. D.; Ok Y. S.; Tong Y. W.; Shariati M. A.; Wu S.; Ahring B. K. Current Status of Biogas Upgrading for Direct Biomethane Use: A Review. Renew. Sustain. Energy Rev. 2021, 149, 111343 10.1016/j.rser.2021.111343.

[ref3] Baltrusaitis J.; Luyben W. L. Methane Conversion to Syngas for Gas-to-Liquids (GTL): Is Sustainable CO2 Reuse via Dry Methane Reforming (DMR) Cost Competitive with SMR and ATR Processes?. ACS Sustain. Chem. Eng. 2015, 3 (9), 2100–2111. 10.1021/acssuschemeng.5b00368.

[ref4] de Smit E.; Weckhuysen B. M. The Renaissance of Iron-Based Fischer–Tropsch Synthesis: On the Multifaceted Catalyst Deactivation Behaviour. Chem. Soc. Rev. 2008, 37 (12), 2758–2781. 10.1039/B805427D. 19020686

[ref5] Cheng W. H.; Kung H. H. Methanol Production and Uses; Marcel Dekker, 1994.

[ref6] Sorenson S. C. Dimethyl Ether in Diesel Engines: Progress and Perspectives. J. Eng. Gas Turbines Power 2001, 123 (3), 652–658. 10.1115/1.1370373.

[ref7] Olah G. A.; Goeppert A.; Prakash G. K. S. Beyond Oil and Gas: The Methanol Economy; Wiley VCH: Weinheim, 2009; Vol. 44, pp 2636–2639. 10.1002/9783527627806.Angew. Chem., Int. Ed. 15800867

[ref8] Olah G. A.; Mo Y. Electrophilic Reaction at Single Bonds. XIII. Chlorination and Chlorolysis of Alkanes in Antimony Pentafluoride-Chlorine-Fluorosulfuryl Chloride Solution at Low Temperature. J. Am. Chem. Soc. 1972, 94 (19), 6864–6865. 10.1021/ja00774a054.

[ref9] Bucsi I.; Olah G. A. Selective Monochlorination of Methane over Solid Acid and Zeolite Catalysts. Catal. Lett. 1992, 16 (1–2), 27–38. 10.1007/bf00764351.

[ref10] Lersch P.; Bandermann F. Conversion of Chloromethane over Metal-Exchanged ZSM-5 to Higher Hydrocarbons. Appl. Catal. 1991, 75 (1), 133–152. 10.1016/S0166-9834(00)83129-2.

[ref11] Jaumain D.; Su B.-L. Direct Catalytic Conversion of Chloromethane to Higher Hydrocarbons over a Series of ZSM-5 Zeolites Exchanged with Alkali Cations. J. Mol. Catal. A 2003, 197 (1–2), 263–273. 10.1016/S1381-1169(02)00622-2.

[ref12] Noronha L. A.; Souza-Aguiar E. F.; Mota C. J. A. Conversion of Chloromethane to Light Olefins Catalyzed by ZSM-5 Zeolites. Catal. Today 2005, 101, 9–13. 10.1016/j.cattod.2004.12.004.

[ref13] Olah G. A.; Gupta B.; Felberg J. D.; Ip W. M.; Husain A.; Karpeles R.; Lammertsma K.; Melhotra A. K.; Trivedi N. J. Electrophilic Reactions at Single Bonds. 20. Selective Monohalogenation of Methane over Supported Acidic or Platinum Metal Catalysts and Hydrolysis of Methyl Halides over. Gamma.-Alumina-Supported Metal Oxide/Hydroxide Catalysts. A Feasible Path for the Oxidative Conversion of Methane into Methyl Alcohol/Dimethyl Ether. J. Am. Chem. Soc. 1985, 107 (24), 7097–7105. 10.1021/ja00310a057.

[ref14] Periana R. A.; Taube D. J.; Gamble S.; Taube H.; Satoh T.; Fujii H. Platinum Catalysts for the High-Yield Oxidation of Methane to a Methanol Derivative. Science 1998, 280 (5363), 560–564. 10.1126/science.280.5363.560. 9554841

[ref15] Zhou X.-P.; Yilmaz A.; Yilmaz G. A.; Lorkovic I. M.; Laverman L. E.; Weiss M.; Sherman J. H.; McFarland E. W.; Stucky G. D.; Ford P. C. An Integrated Process for Partial Oxidation of Alkanes. Chem. Commun. 2003, 2294, 2294 10.1039/b307235e. 14518881

[ref16] Xu H. F.; Wang K. X.; Li W. S.; Zhou X. P. Dimethyl Ether Synthesis from Methane by Non Syngas Process. Catal. Lett. 2005, 100 (1–2), 53–57. 10.1007/s10562-004-3085-x.

[ref17] Khaleel A.; Shehadi I.; Al-Marzouqi A. Catalytic Conversion of Chloromethane to Methanol and Dimethyl Ether over Mesoporous γ-Alumina. Fuel Proc. Technol. 2011, 92 (9), 1783–1789. 10.1016/j.fuproc.2011.04.029.

[ref18] Fernandes D. R.; Rosenbach N.; Mota C. J. A. Catalytic Conversion of Chloromethane to Methanol and Dimethyl Ether over Metal-Exchanged Zeolite Y. Appl. Catal., A 2009, 367 (1–2), 108–112. 10.1016/j.apcata.2009.07.043.

[ref19] Fernandes D.; Leite T.; Mota C. Catalytic Conversion of Chloromethane to Methanol and Dimethyl Ether over Two Catalytic Beds: A Study of Acid Strength. Braz. J. Petrol. Gas 2010, 4 (3), 083 10.5419/bjpg2010-0009.

[ref20] Murray D. K.; Chang J. W.; Haw J. F. Conversion of Methyl Halides to Hydrocarbons on Basic Zeolites: A Discovery by in Situ NMR. J. Am. Chem. Soc. 1993, 115 (11), 4732–4741. 10.1021/ja00064a037.

[ref21] Corrêa R. J.; Mota C. J. A Fast and Easy Computational Method to Calculate the 13C NMR Chemical Shift of Organic Species Adsorbed on the Zeolite Surface. J. Am. Chem. Soc. 2002, 124 (14), 3484–3485. 10.1021/ja012583y. 11929219

[ref22] Wang W.; Hunger M. Reactivity of Surface Alkoxy Species on Acidic Zeolite Catalysts. Acc. Chem. Res. 2008, 41 (8), 895–904. 10.1021/ar700210f. 18605741

[ref23] Minova I. B.; Matam S. K.; Greenaway A.; Catlow C. R. A.; Frogley M. D.; Cinque G.; Wright P. A.; Howe R. F. Elementary Steps in the Formation of Hydrocarbons from Surface Methoxy Groups in HZSM-5 Seen by Synchrotron Infrared Microspectroscopy. ACS Catal. 2019, 9 (7), 6564–6570. 10.1021/acscatal.9b01820.

[ref24] Ivanova I. I.; Corma A. Surface Species Formed and Their Reactivity during the Alkylation of Toluene by Methanol and Dimethyl Ether on Zeolites as Determined by in Situ 13C MAS NMR. J. Phys. Chem. B 1997, 101 (4), 547–551. 10.1021/jp961468k.

[ref25] Wang W.; Seiler M.; Hunger M. Role of Surface Methoxy Species in the Conversion of Methanol to Dimethyl Ether on Acidic Zeolites Investigated by in Situ Stopped-Flow MAS NMR Spectroscopy. J. Phys. Chem. B 2001, 105 (50), 12553–12558. 10.1021/jp0129784.

[ref26] Xu M.; Lunsford J. H.; Goodman D. W.; Bhattacharyya A. Synthesis of Dimethyl Ether (DME) from Methanol over Solid-Acid Catalysts. Appl. Catal., A 1997, 149 (2), 289–301. 10.1016/S0926-860X(96)00275-X.

[ref27] Olsbye U.; Saure O. V.; Muddada N. B.; Bordiga S.; Lamberti C.; Nilsen M. H.; Lillerud K. P.; Svelle S. Methane Conversion to Light Olefins - How Does the Methyl Halide Route Differ from the Methanol to Olefins (MTO) Route?. Catal. Today 2011, 171, 211–220. 10.1016/j.cattod.2011.04.020.

[ref28] Jiang Z.; Shen B.; Zhao J.; Wang L.; Kong L.; Xiao W. Enhancement of Catalytic Performances for the Conversion of Chloromethane to Light Olefins over SAPO-34 by Modification with Metal Chloride. Ind. Eng. Chem. Res. 2015, 54 (49), 12293–12302. 10.1021/acs.iecr.5b03586.

[ref29] Dapprich S.; Komáromi I.; Byun K. S.; Morokuma K.; Frisch M. J. A New ONIOM Implementation in Gaussian98. Part I. The Calculation of Energies, Gradients, Vibrational Frequencies and Electric Field Derivatives. J. Mol. Struct.: Theochem 1999, 461, 1–21. 10.1016/S0166-1280(98)00475-8.

[ref30] Sillar K.; Burk P. Hybrid Quantum Chemical and Density Functional Theory (ONIOM) Study of the Acid Sites in Zeolite ZSM-5. J. Phys. Chem. B 2004, 108 (28), 9893–9899. 10.1021/jp037770s.

[ref31] Sillar K.; Burk P. Calculation of the Properties of Acid Sites of the Zeolite ZSM-5 Using ONIOM Method. J. Mol. Struct.: THEOCHEM 2002, 589–590, 281–290. 10.1016/S0166-1280(02)00283-X.

[ref32] McCusker L. B.; Olson D. H.; Baerlocher C. Atlas of Zeolite Framework Types, 6th ed.; Elsevier Science: London, England, 2007. 10.1016/B978-0-444-53064-6.X5186-X.

[ref33] Jirak Z.; Vratislav S.; Bosáček V. A Neutron Diffraction Study of H, Na-Y Zeolites. J. Phys. Chem. Solids 1980, 41 (10), 1089–1095. 10.1016/0022-3697(80)90064-5.

[ref34] Hill J.-R.; Freeman C. M.; Delley B. Bridging Hydroxyl Groups in Faujasite: Periodic vs Cluster Density Functional Calculations. J. Phys. Chem. A 1999, 103 (19), 3772–3777. 10.1021/jp990031k.

[ref35] Fritsch M.; Trucks G.; Cheeseman J.; Scalmani G.; Clemente F.; Hratchian H.; Caricato M.; Izmaylov A.; Hess J.; Fox A.; Gaussian 09. Revision C. 3: Pittsburgh, PA, 2009.

[ref36] Rosenbach N. Jr.; Mota C. J. A. A DFT–ONIOM Study on the Effect of Extra-Framework Aluminum on USY Zeolite Acidity. Appl. Catal., A 2008, 336, 54–60. 10.1016/j.apcata.2007.09.048.

[ref37] Rosenbach N. Jr.; dos Santos A. P.; Franco M.; Mota C. J. The Tert-Butyl Cation on Zeolite Y: A Theoretical and Experimental Study. Chem. Phys. Lett. 2010, 485 (1–3), 124–128. 10.1016/j.cplett.2009.12.003.

[ref38] Grimme S.; Antony J.; Ehrlich S.; Krieg H. A consistent and accurate ab initio parametrization of density functional dispersion correction (DFT-D) for the 94 elements H-Pu. J. Chem. Phys. 2010, 132 (15), 154104 10.1063/1.3382344. 20423165

[ref39] Zicovich-Wilson C.; Viruela P.; Corma A. Formation of Surface Methoxy Groups on H-Zeolites from Methanol. A Quantum Chemical Study. J. Phys. Chem. 1995, 99 (35), 13224–13231. 10.1021/j100035a029.

[ref40] Wang Z.; Chen X.-F. A Periodic Density Functional Theory Study on Methanol Adsorption in HSAPO-34 Zeolites. Chem. Phys. Lett. 2021, 771, 138532 10.1016/j.cplett.2021.138532.

[ref41] Rosenbach N.; Mota C. J. A. Carbocation inside the Cage: A Periodical Dft Study on the Interaction of the C4H7+system with Alkali Metal Ion-Exchanged Zeolite y. Arkivoc 2020, 2020 (2), 102–112. 10.24820/ark.5550190.p011.113.

[ref42] Carey F. A.; Sundberg R. J. Advanced Organic Chemistry; Springer US: Boston, MA, 1990. 10.1007/978-1-4613-9797-7.

[ref43] Jones A. J.; Iglesia E. Kinetic, Spectroscopic, and Theoretical Assessment of Associative and Dissociative Methanol Dehydration Routes in Zeolites. Angew. Chem. Internat. Ed. 2014, 53 (45), 12177–12181. 10.1002/anie.201406823. 25212869

[ref44] Franco M.; Rosenbach N.; Ferreira G. B.; Guerra A. C. O.; Kover W. B.; Turci C. C.; Mota C. J. A.; Rearrangement C. J. A. Rearrangement, Nucleophilic Substitution, and Halogen Switch Reactions of Alkyl Halides over NaY Zeolite: Formation of the Bicyclobutonium Cation Inside the Zeolite Cavity. J. Am. Chem. Soc. 2008, 130 (5), 1592–1600. 10.1021/ja0742939. 18189387

[ref45] Henrique F. J.; Barbosa P. A.; Grisolia L. M.; Silva R. J.; Mota C. J. Evidence for Supramolecular Catalysis in the Synthesis of Cyclic Organic Carbonates over Zeolite Y Impregnated with Metal Iodides. Mol. Catal. 2022, 522, 112216 10.1016/j.mcat.2022.112216.

[ref46] de Andrade K. N.; Peixoto B. P.; Carneiro J. W. d. M.; Fiorot R. G. Exploring borderline S_N_1–S_N_2 mechanisms: the role of explicit solvation protocols in the DFT investigation of isopropyl chloride. RSC Adv. 2024, 14, 4692–4701. 10.1039/D4RA00066H. 38318615 PMC10841197

