# Supplementary material for: Zeolites as Solid Solvents: Explaining the Chloromethane Hydrolysis over Metal-Exchanged Zeolite Y by DFT Calculations
Source: ACS Omega. 2024 Sep 21;9(39):40522–30. doi: 10.1021/acsomega.4c03593 (PMC11447861; doi:10.1021/acsomega.4c03593)
Supplement: Supplementary file 1 — ao4c03593_si_001.pdf [file ao4c03593_si_001.pdf]

**SUPPORTING INFORMATION**

**Zeolites as Solid Solvents: Explaining the Chloromethane Hydrolysis over Metal-Exchanged Zeolite Y by DFT Calculations**

Nilton Rosenbach Jr,[1] Daniella R. Fernandes[2] and Claudio J. A. Mota[2,3,4]*

[1]*Universidade do Estado do Rio de Janeiro, Faculdade de Ciência Exatas e Engenharias, Av. Manuel Caldeira de Alvarenga, 1203, 23070-200, Rio de Janeiro, Brazil.*

[2] *Universidade Federal do Rio de Janeiro, Instituto de Química. Av Athos da Silveira Ramos 149, CT Bloco A, 21941-909, Rio de Janeiro, Brazil.*

[3] *Universidade Federal do Rio de Janeiro, Escola de Química. Av Athos da Silveira Ramos 149, CT Bloco E, 21941-909, Rio de Janeiro, Brazil.*

[4] *INCT Energia & Ambiente, UFRJ, 21941-909, Rio de Janeiro, Brazil.*

*cmota@iq.ufrj.br*

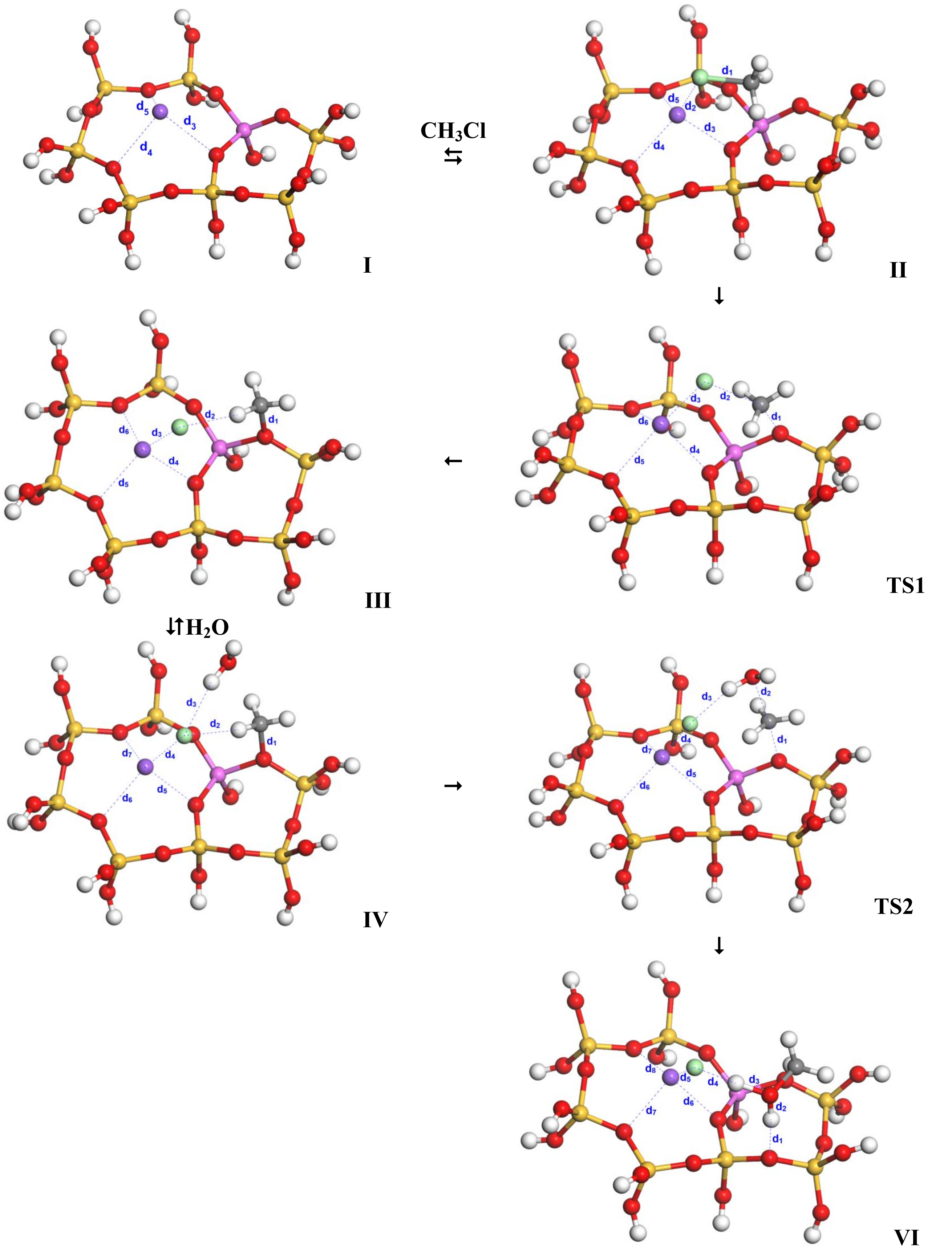


**Figure S1:** Optimized structures of all species within the the alkoxide mechanism for chloromethane hydrolysis catalyzed by zeolite Y exchanged with sodium (for clarity, only higher layer atoms are shown).

**Table S1:** The optimized structural parameters for Cluster I and Adsorption Complex II, where the geometric parameters ($d_1$, $d_2$, $d_3$, $d_4$, and $d_5$) are referenced to Figure S1 (values in Angstrom).

| Parameter | I | | | | | II | | | | |
|---|---|---|---|---|---|---|---|---|---|---|
| | Li | Na | K | Mg[a] | Mg[b] | Li | Na | K | Mg[a] | Mg[b] |
| $d_1$ | - | - | - | - | - | 1.82 | 1.82 | 1.81 | 1.83 | 1.83 |
| $d_2$ | - | - | - | - | - | 2.42 | 2.81 | 3.21 | 2.47 | 2.48 |
| $d_3$ | 1.91 | 2.22 | 2.71 | 2.02 | 2.05 | 1.95 | 2.30 | 2.70 | 2.10 | 2.10 |
| $d_4$ | 2.43 | 2.35 | 2.87 | 2.01 | 2.10 | 2.71 | 2.55 | 2.93 | 2.03 | 2.15 |
| $d_5$ | 2.03 | 2.29 | 2.80 | 2.06 | 1.97 | 2.05 | 2.38 | 2.81 | 2.11 | 1.99 |

Al atoms in the relative position: (a) 1-3 and (b) 1-4

**Table S2:** The optimized structural parameters for the Transition State TS1 and the alkoxide intermediate III, where the geometric parameters $d_1$, $d_2$, $d_3$, and $d_4$, $d_5$, and $d_6$ are referenced to Figure S1 (values in Angstrom).

| Parameter | TS1 | | | | | III | | | | |
|---|---|---|---|---|---|---|---|---|---|---|
| | Li | Na | K | Mg[a] | Mg[b] | Li | Na | K | Mg[a] | Mg[b] |
| $d_1$ | 1.96 | 1.89 | 1.81 | 2.07 | 2.08 | 1.51 | 1.52 | 1.52 | 1.50 | 1.51 |
| $d_2$ | 2.57 | 2.61 | 2.64 | 2.49 | 2.49 | 2.47 | 2.13 | 2.17 | 2.46 | 2.61 |
| $d_3$ | 2.26 | 2.60 | 2.94 | 2.37 | 2.38 | 2.13 | 2.49 | 2.85 | 2.24 | 2.24 |
| $d_4$ | 2.16 | 2.53 | 3.07 | 2.25 | 2.24 | 2.41 | 2.55 | 3.06 | 2.40 | 2.40 |
| $d_5$ | 2.54 | 2.68 | 2.99 | 2.05 | 2.22 | 2.26 | 2.69 | 2.99 | 2.05 | 2.20 |
| $d_6$ | 2.43 | 2.58 | 2.95 | 2.19 | 2.03 | 2.16 | 2.57 | 2.96 | 2.20 | 2.04 |

Al atoms in the relative position: (a) 1-3 and (b) 1-4

**Table S3:** The optimized structural parameters for the Adsorption Complex IV, where the geometric parameters $d_1$, $d_2$, $d_3$, and $d_4$, $d_5$, $d_6$, and $d_7$ are referenced to Figure S1 (values in Angstrom).

| Parameter | Counter-ion | | | | |
|---|---|---|---|---|---|
| | Li | Na | K | Mg[a] | Mg[b] |
| $d_1$ | 1.52 | 1.53 | 1.54 | 1.52 | 1.50 |
| $d_2$ | 2.96 | 2.49 | 2.42 | 2.78 | 2.73 |
| $d_3$ | 2.10 | 2.22 | 2.22 | 2.42 | 2.39 |
| $d_4$ | 2.19 | 2.48 | 2.84 | 2.25 | 2.24 |
| $d_5$ | 2.27 | 2.51 | 2.96 | 2.38 | 3.29 |
| $d_6$ | 2.39 | 2.59 | 2.96 | 2.04 | 2.69 |
| $d_7$ | 2.17 | 2.53 | 2.93 | 2.20 | 2.06 |

Al atoms in the relative position: (a) 1-3 and (b) 1-4

**Table S4:** The optimized structural parameters for the transition state TS2, where the geometric parameters $d_1$, $d_2$, $d_3$, and $d_4$, $d_5$, $d_6$, $d_7$, and $d_8$ are referenced to Figure S1 (values in Angstrom).

| Parameter | TS2 | | | | | VI | | | | |
|---|---|---|---|---|---|---|---|---|---|---|
| | **Li** | **Na** | **K** | **Mg$^a$** | **Mg$^b$** | **Li** | **Na** | **K** | **Mg$^a$** | **Mg$^b$** |
| $d_1$ | 1.90 | 1.91 | 1.90 | 1.98 | 1.98 | 1.81 | 1.86 | 1.95 | 1.67 | 1.40 |
| $d_2$ | 2.13 | 2.13 | 2.15 | 2.04 | 2.04 | 0.99 | 0.99 | 0.99 | 1.01 | 1.08 |
| $d_3$ | 1.99 | 1.99 | 1.98 | 2.08 | 2.08 | 1.20 | 1.25 | 1.32 | 1.07 | 1.04 |
| $d_4$ | 2.19 | 2.52 | 2.88 | 2.29 | 2.09 | 1.59 | 1.53 | 1.48 | 1.78 | 1.89 |
| $d_5$ | 2.10 | 2.45 | 2.88 | 2.26 | 2.26 | 2.28 | 2.63 | 3.06 | 2.33 | 2.31 |
| $d_6$ | 2.61 | 2.56 | 2.93 | 2.04 | 2.51 | 2.04 | 2.35 | 2.75 | 2.21 | 2.23 |
| $d_7$ | 2.10 | 2.46 | 2.90 | 2.19 | 2.03 | 2.97 | 2.63 | 3.00 | 2.06 | 2.20 |
| $d_8$ | - | - | - | - | - | 2.08 | 2.38 | 2.80 | 2.14 | 2.02 |

Al atoms in the relative position: (a) 1-3 and (b) 1-4

**Table S5:** The optimized structural parameters for the Adsorption Complex V and the Transition State TS3, where the geometric parameters $d_1$, $d_2$, $d_3$, $d_4$, $d_5$, $d_6$, $d_7$, and $d_8$ are referenced to Figure S2 (values in Angstrom).

| Parameter | V | | | | | TS3 | | | | |
|---|---|---|---|---|---|---|---|---|---|---|
| | **Li** | **Na** | **K** | **Mg$^a$** | **Mg$^b$** | **Li** | **Na** | **K** | **Mg$^a$** | **Mg$^b$** |
| $d_1$ | 2.40 | 2.82 | 3.30 | 2.45 | 2.46 | 1.65 | 1.64 | 1.66 | 1.80 | 1.79 |
| $d_2$ | 4.69 | 2.40 | 2.81 | 6.06 | 6.26 | 1.02 | 1.02 | 1.02 | 1.00 | 1.00 |
| $d_3$ | 1.94 | 2.33 | 2.75 | 2.09 | 2.08 | 1.83 | 1.82 | 1.81 | 1.98 | 2.00 |
| $d_4$ | 2.73 | 3.02 | 3.20 | 2.04 | 2.18 | 2.54 | 2.54 | 2.56 | 2.37 | 2.36 |
| $d_5$ | 2.04 | 2.55 | 2.93 | 2.12 | 1.99 | 2.22 | 2.54 | 2.90 | 2.33 | 2.34 |
| $d_6$ | - | - | - | - | - | 2.05 | 2.39 | 2.83 | 2.19 | 2.19 |
| $d_7$ | - | - | - | - | - | 2.39 | 2.50 | 2.90 | 2.04 | 2.18 |
| $d_8$ | - | - | - | - | - | 2.19 | 2.45 | 2.89 | 2.17 | 2.02 |

Al atoms in the relative position: (a) 1-3 and (b) 1-4

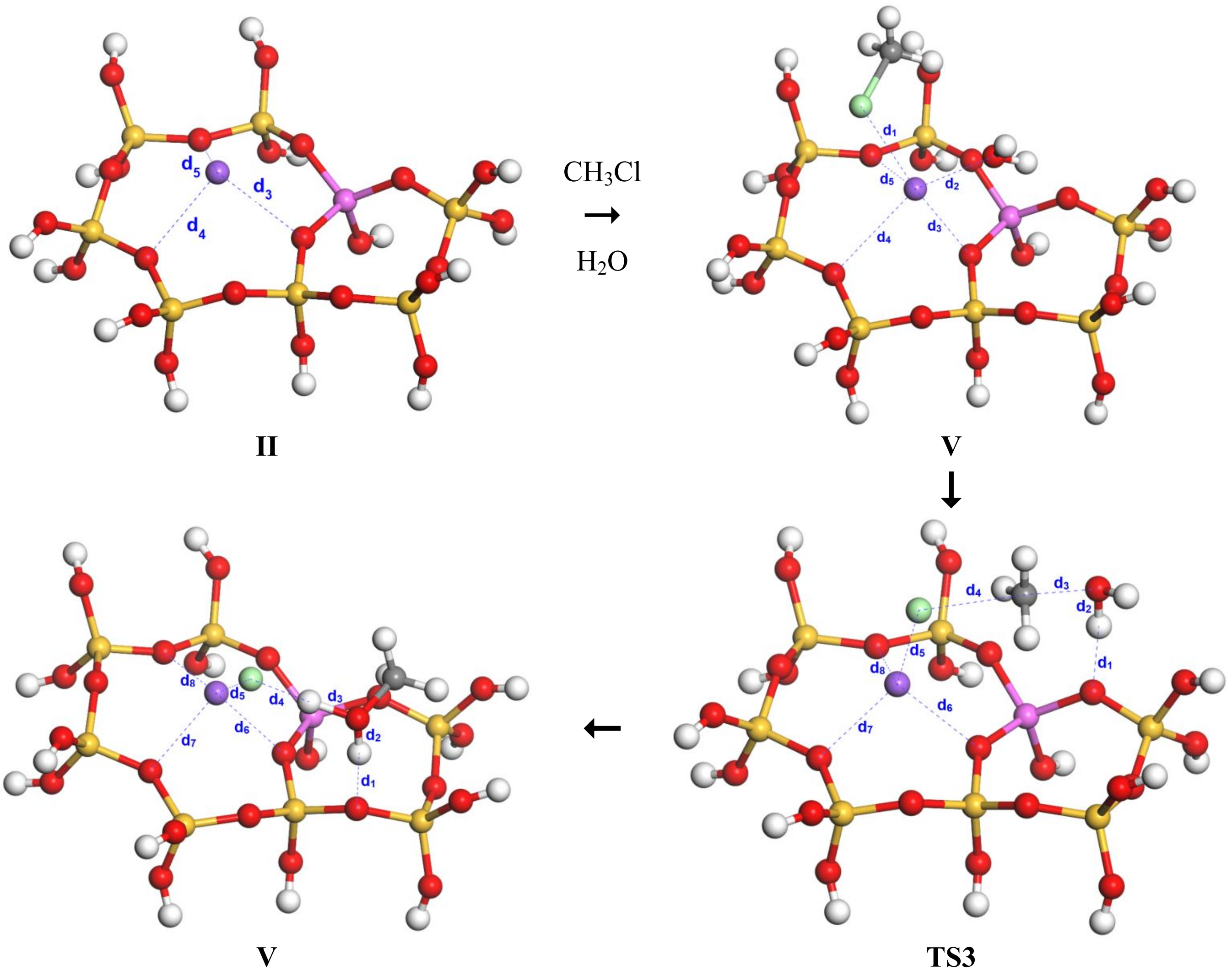


**Figure S2:** Optimized structures of all species within the direct mechanism for chloromethane hydrolysis catalyzed by zeolite Y exchanged with sodium (for clarity, only higher layer and link atoms are shown).

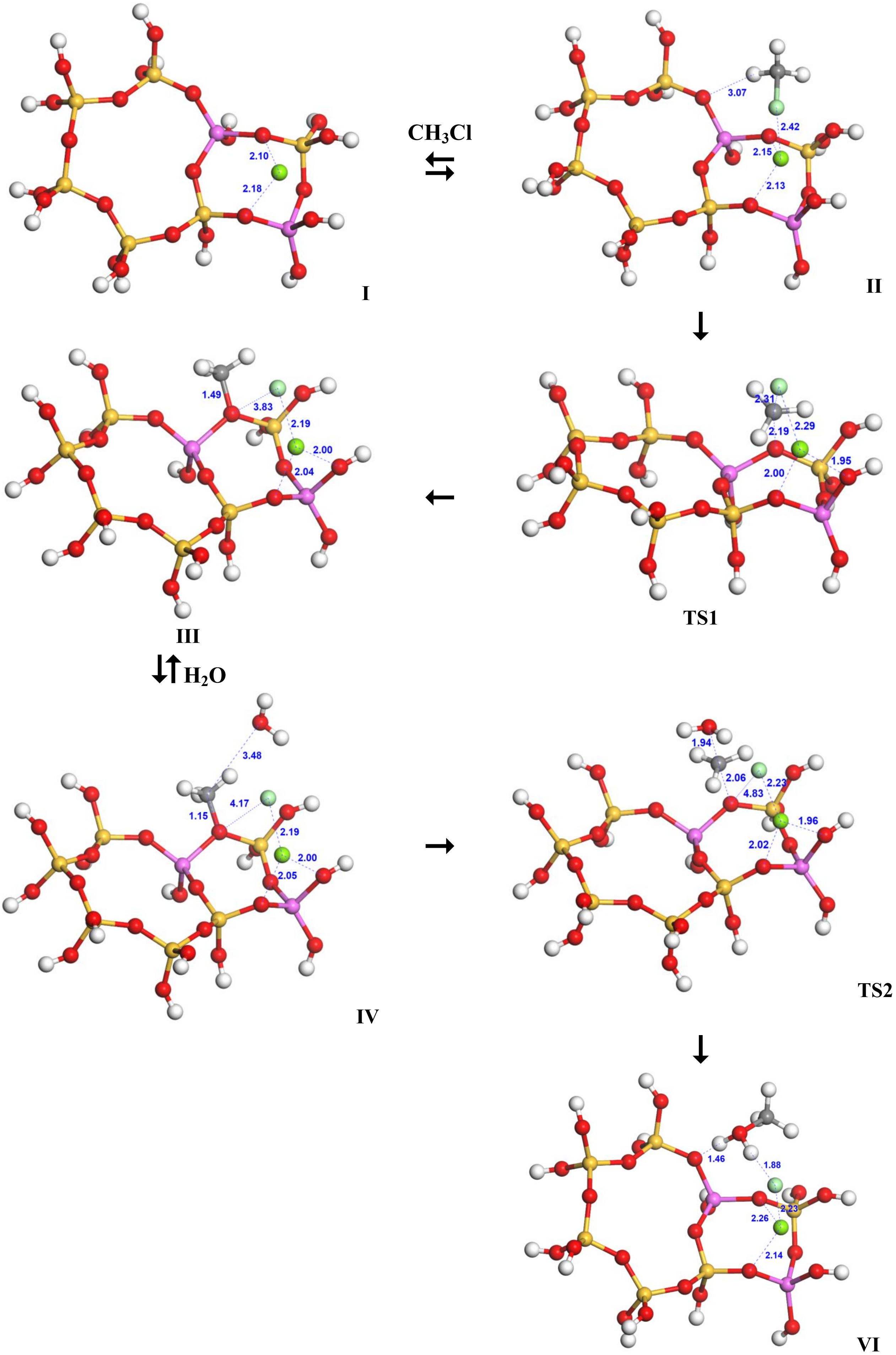
I
2.10
2.18
CH3Cl
II
3.07
2.42
2.15
2.13
TS1
2.31
2.19
2.29
1.95
2.00
III
1.49
3.83
2.19
2.00
2.04
H2O
IV
3.48
1.15
4.17
2.19
2.00
2.05
TS2
1.94
2.06
2.23
4.83
1.96
2.02
VI
1.46
1.88
2.23
2.26
2.14

**Figure S3:** Optimized structures of all species within the alkoxide mechanism for chloromethane hydrolysis catalyzed by zeolite Y exchanged with magnesium with the Al atoms in the relative position 1-2 (for clarity, only higher layer and link atoms are shown).

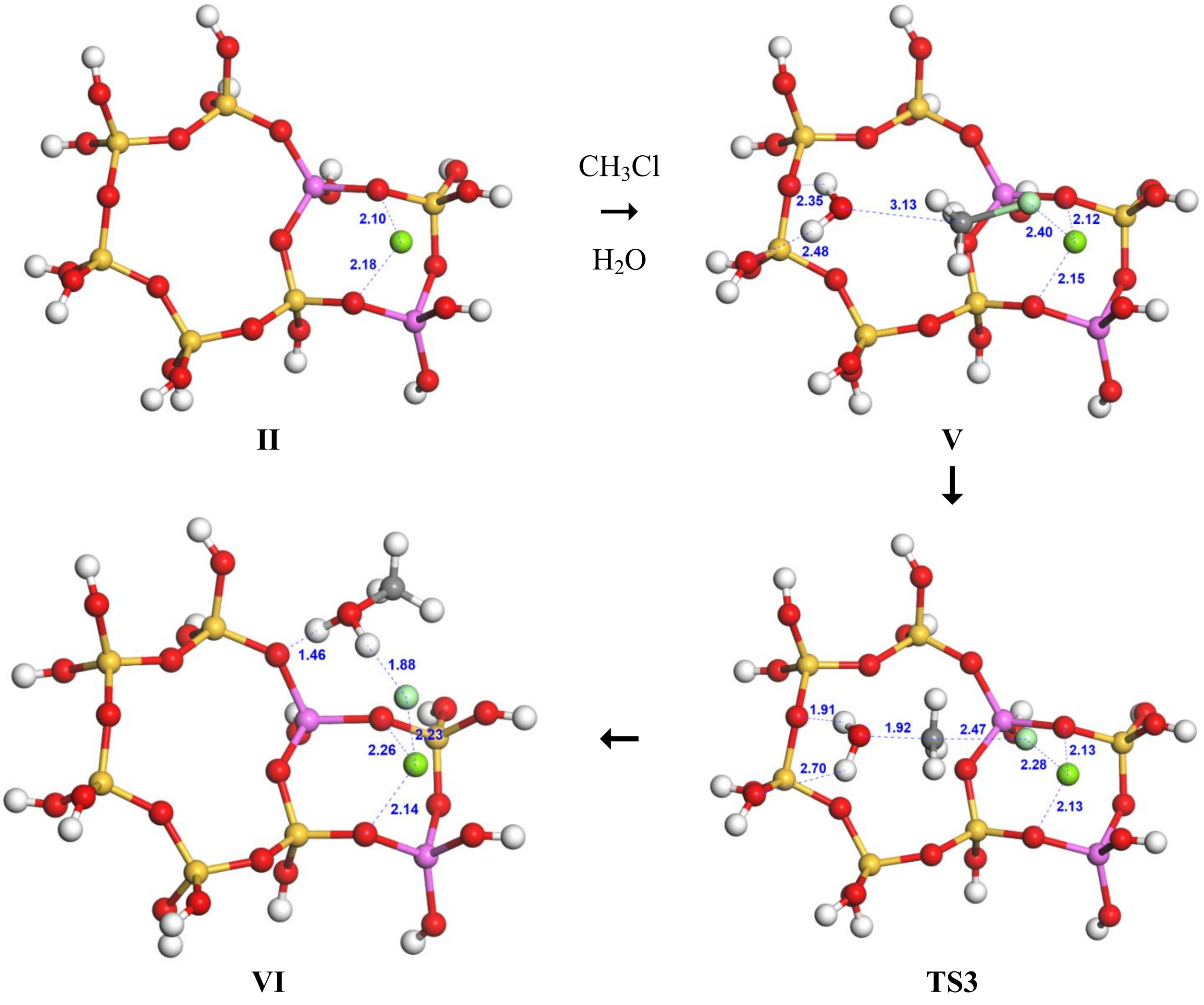


**Figure S4:** Optimized structures of all species within direct the mechanism for chloromethane hydrolysis catalyzed by zeolite Y exchanged with magnesium with the Al atoms in the relative position 1-2 (for clarity, only higher layer and link atoms are shown).

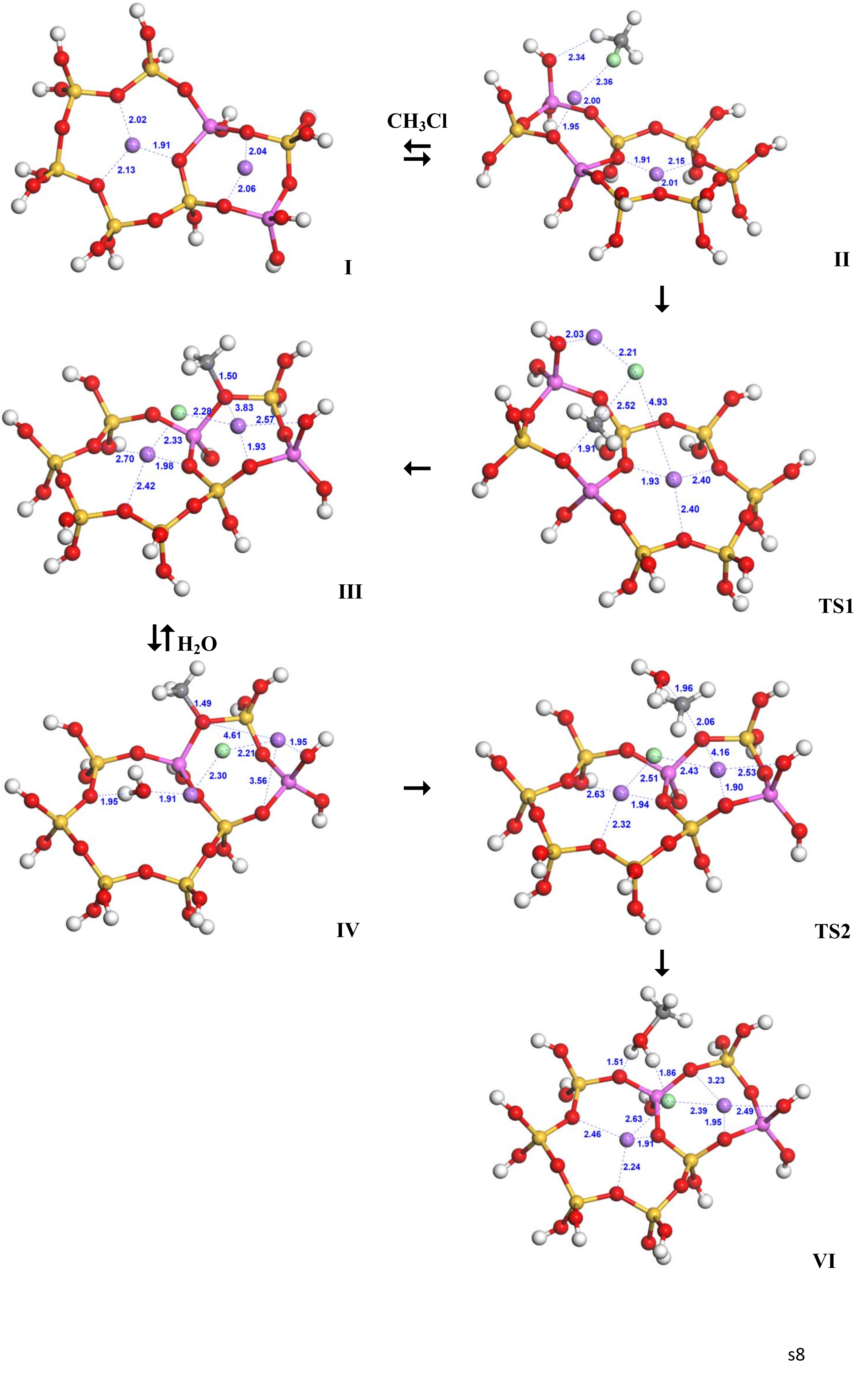
2.02
1.91
2.04
2.13
2.06
I
$CH_3Cl$
2.34
2.36
2.00
1.95
1.91
2.15
2.01
II
1.50
2.28
3.83
2.57
2.33
2.70
1.98
1.93
2.42
III
2.03
2.21
2.52
4.93
1.91
1.93
2.40
2.40
TS1
$H_2O$
1.49
4.61
2.21
1.95
2.30
3.56
1.95
1.91
IV
1.96
2.06
4.16
2.43
2.53
2.51
2.63
1.94
1.90
2.32
TS2
1.51
1.86
3.23
2.63
2.39
2.49
1.95
2.46
1.91
2.24
VI

**Figure S5:** Optimized structures of all species within the alkoxide mechanism for chloromethane hydrolysis catalyzed by zeolite Y exchanged with lithium and two Al atoms in the relative position 1-2 (for clarity, only higher layer and link atoms are shown).

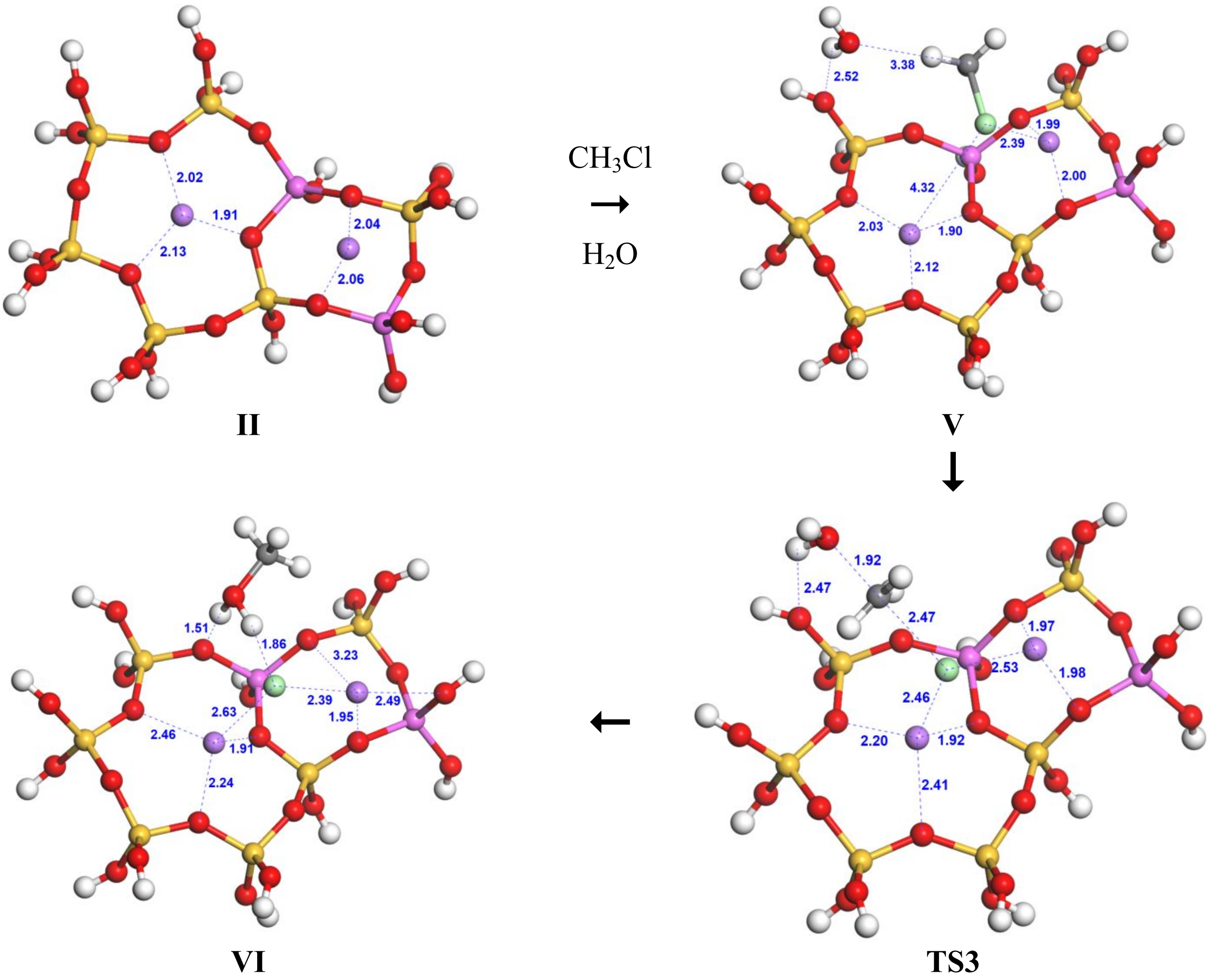


**Figure S6:** Optimized structures of all species within the direct mechanism for chloromethane hydrolysis catalyzed by zeolite Y exchanged with lithium and two Al atoms in the relative position 1-2 (for clarity, only higher layer and link atoms are shown).

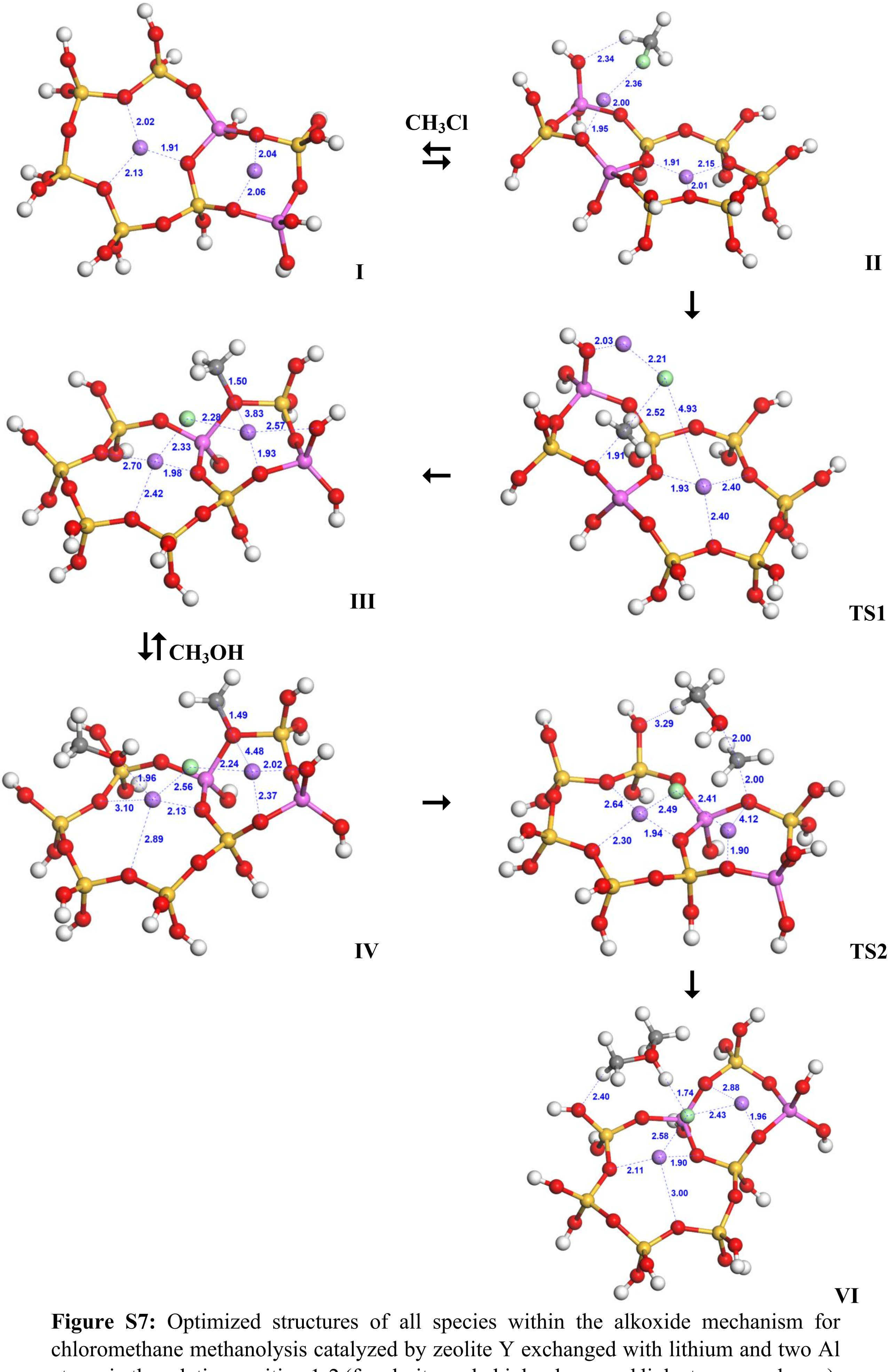


**Figure S7:** Optimized structures of all species within the alkoxide mechanism for chloromethane methanolysis catalyzed by zeolite Y exchanged with lithium and two Al atoms in the relative position 1-2 (for clarity, only higher layer and link atoms are shown).

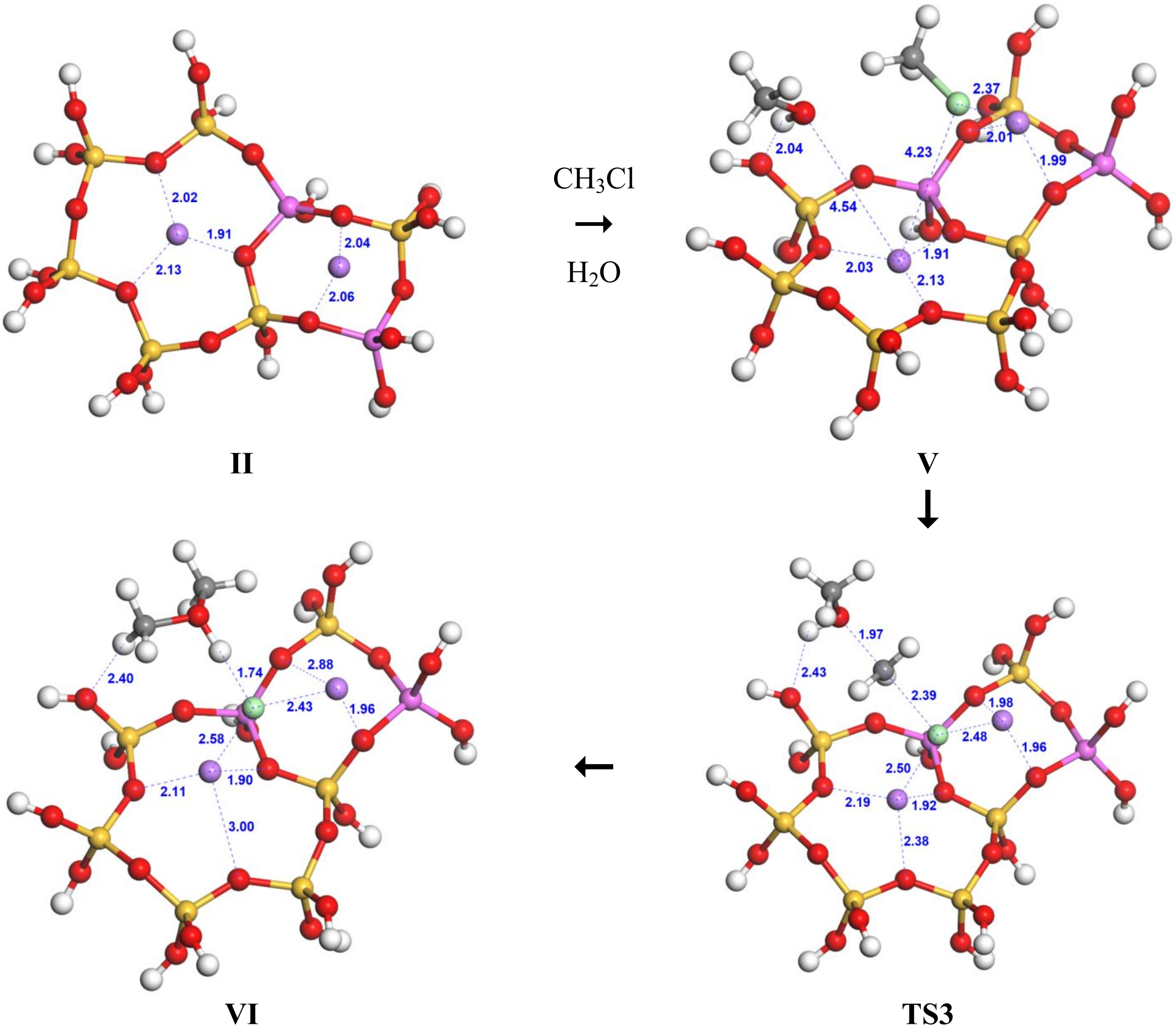


**Figure S8:** Optimized structures of all species within the direct mechanism for chloromethane methanolysis catalyzed by zeolite Y exchanged with lithium and two Al atoms in the relative position 1-2 (for clarity, only higher layer and link atoms are shown).

**Table S6:** Calculated energy profile of the hydrolysis of chloromethane over metal-exchanged Y zeolites considering the alkoxide and the direct route. The energy values (in kJ $mol^{-1}$) refer to the barriers and energy differences displayed in Figure 3. The first, second and third values were calculated using electronic energy determined at ONIOM(M062X:PM6), M062X and ONIOM(MP2:M062X) levels and 6-31++G(d,p) basis set, respectively.

| Ion | $E_{ads}$ | Alkoxide Route (a) | | | Direct Route (a) | $\Delta H_{total}$ |
|---|---|---|---|---|---|---|
| | | Ea1 | ΔH1 | Ea2 | Ea3 | |
| *Hydrolysis* | | | | | | |
| $Li^{+}$(b) | -68.4/-69.6/69.2 | 163.1/143.9/192.7 | -64.3/-55.0/-58.8 | 26.9/28.4/54.0 | 96.2/78.6/103.6 | -148.9/-139.5/-134.6 |
| $Li^{+}$(c) | -77.2/-73.3/-75.8 | 99.3/89.5/142.8 | -67.2/-69.5/-73.6 | 114.3/115.6/141.2 | 59.7/60.1/85.2 | -144.6/-166.9/-154.4 |
| $Na^{+}$(b) | -68.6/-65.7/-67.1 | 141.2/132.3/177.6 | -42.8/-38.1/-41.0 | 28.3/20.3/41.3 | 112.5/117.3/140.0 | -156.1/-134.0/-140.6 |
| $K^{+}$(b) | -58.4/-46.4/-47.0 | 147.8/126.4/175.1 | -25.2/-27.6/-30.6 | 26.2/23.2/45.2 | 131.8/142.7/167.5 | -163.7/-118.1/-122.3 |
| $Mg^{2+}$(c) | -133.7/-135.4/-136.9 | 115.6/98.8/137.9 | -51.0/-24.4/-21.6 | 90.3/73.6/92.7 | 58.6/38.0/65.4 | -191.8/-253.8/-173.8 |
| $Mg^{2+}$(d) | -127.4/-105.5/-105.2 | 121.3/127.7/174.4 | -61.3/-41.4/-46.2 | 48.3/40.4/65.9 | 57.7/43.9/73.6 | -206.7/-179.7/-180.2 |
| $Mg^{2+}$(e) | -125.5/-109.1/106.8 | 119.9/120.6/167.5 | -103.2/-76.6/-76.4 | 90.0/76.5/99.7 | 55.3/39.4/68.7 | -219.8/-217.7/-177.4 |
| *Methanolysis* | | | | | | |
| Li3-$Li^{+}$(c) | -77.2/-73.3/-75.8 | 99.3/89.5/142.8 | -108.5/-78.9/-74.4 | 108/104.5/119.8 | 88.2/53.2/78.7 | -151.6/-151.7/-98.6 |
| Mg5-$Mg^{2+}$(c) | -133.7/-135.4/-136.9 | 115.6/98.8/137.9 | -42.3/-25.6/-23.8 | 58.8/71.6/70.0 | 54.5/24.1/57.5 | -217.9/-234.9/-218.8 |

(a) Referred to figure 2; (b) Calculated with 1 Al atom; (c) Calculated with 2 Al atoms at 1,2 relative positions (figure 1);
(d) Calculated with 2 Al atoms at 1,3 relative positions (figure 1); (e) Calculated with 2 Al atoms at 1,4 relative positions (figure 1).

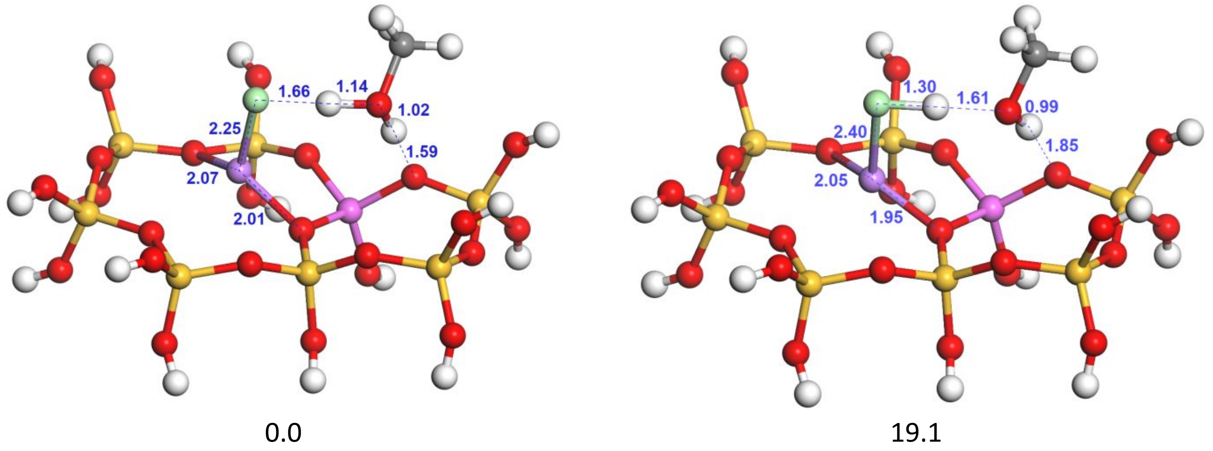


**Figure S9:** Optimized structures for protonated and (left) and deprotonated (right) methanol species on zeolite Y exchanged with lithium (for clarity, only higher layer and link atoms are shown). Relative energy between both species in kJ $mol^{-1}$.
